# Sociodemographic factors associated with suicidal behavior at a federal public university in the Western Brazilian Amazon

**DOI:** 10.1590/0034-7167-2023-0102

**Published:** 2023-12-08

**Authors:** Maria Aline do Nascimento Oliveira, Evandro Piccinelli da Silva, Aristeia Nunes Sampaio, Isabela Saura Sartoreto Mallagoli, Dulce Aparecida Barbosa, Thiago da Silva Domingos, Angélica Gonçalves Silva Belasco

**Affiliations:** I Universidade Federal de São Paulo. São Paulo, São Paulo, Brazil; II Universidade Federal do Acre. Cruzeiro do Sul, Acre, Brazil; III Universidade Federal do Acre. Rio Branco, Acre, Brazil

**Keywords:** Public Health, Self-Injurious Behavior, Risk Factors, Sociodemographic Factors, Mental Health, Factores Sociodemográficos, Factores de Riesgo, Salud Mental, Asunción de Riegos, Conducta Autodestructiva, Fatores Sociodemográficos, Suicídio, Universidades, Fatores de Risco, Saúde Mental

## Abstract

**Objective:**

to determine risk factors for suicidal behavior among students and employees of a federal public university in the Brazilian Western Amazon.

**Methods:**

an analytical cross-sectional study of survey and association between variables with a sample of 475 participants. Statistical analyzes were performed using the Mann-Whitney test, Pearson’s chi-square test, likelihood ratio test or Fisher’s exact test and a logistic regression model. A significance level of 5% was used (p-value< 0.05).

**Results:**

a higher proportion of suicidal behavior was found in younger participants, females, who had no religion or had one, but were non-practicing, who did not have children and/or had a monthly family income of less than two minimum wages. Lower proportions of suicidal behavior were identified in heterosexuals and/or married or in a stable relationship.

**Conclusion:**

the study suggests a relationship between sociodemographic factors and suicidal behavior in the studied academic community.

## RESUMO

**Objetivo:**

determinar os fatores de risco para o comportamento suicida entre estudantes e servidores de uma universidade pública federal da Amazônia Ocidental brasileira.

**Métodos:**

estudo do tipo transversal analítico de levantamento e associação entre variáveis com uma amostra de 475 participantes. As análises estatísticas foram feitas por Teste de Mann-Whitney, Teste Qui-Quadrado de Pearson, Teste da Razão de Verossimilhança ou Teste Exato de Fisher e modelo de regressão logística. Foi utilizado um nível de significância de 5% (p-valor < 0,05).

**Resultados:**

foi encontrada maior proporção de comportamento suicida em participante com idade menor, sexo feminino, que não possuía religião ou possuía, mas não era praticante, que não tinha filhos e/ou possuía renda familiar mensal inferior a dois salários mínimos. Menores proporções de comportamento suicida foram identificadas em heterossexuais e/ou casados ou em união estável.

**Conclusão:**

o estudo sugere relação entre fatores sociodemográficos com o comportamento suicida na comunidade acadêmica estudada.

## RESUMEN

**Objetivo:**

determinar los factores de riesgo para la conducta suicida entre estudiantes y empleados de una universidad pública federal en la Amazonía occidental brasileña.

**Métodos:**

estudio analítico transversal de encuesta y asociación entre variables con una muestra de 475 participantes. Los análisis estadísticos se realizaron mediante las pruebas Mann-Whitney, chi-cuadrado de Pearson, razón de verosimilitud o exacta de Fisher y un modelo de regresión logística. Se utilizó un nivel de significancia del 5% (p-valor <0,05).

**Resultados:**

se encontró mayor proporción de conducta suicida en los participantes más jóvenes, mujeres, que no tenían religión o la tenían pero no la practicaban, que no tenían hijos y/o tenían un ingreso familiar mensual inferior a dos salarios mínimos. Se identificaron menores proporciones de conducta suicida en heterosexuales y/o casados o en relación estable.

**Conclusión:**

el estudio sugiere una relación entre factores sociodemográficos y conducta suicida en la comunidad académica estudiada.

## INTRODUCTION

Suicide is a complex public health problem and the result of interaction between psychological, social, biological and environmental factors^(1)^.

According to a report by the World Health Organization (WHO), about 703,000 people died by suicide in the world in 2019, with 13,523 of these occurring in Brazil. It was noticed that the world suicide rates are decreasing, but in the Americas the mortality from this cause is increasing. The global rate decreased by 36% between 2000 and 2019 and in the same period, while in the Americas region, there was an increase of 17%. Suicide appears as the fourth most recurrent cause of death among individuals aged 15 to 29 years, behind traffic accidents, tuberculosis and interpersonal violence^(2)^.

The national suicide rate in 2019 was 6.6 per 100,000 population. When analyzing the distribution of death risk by suicide according to age group among Brazilian regions, it was observed that the South, North and Midwest regions had the highest mortality rates for adolescents aged 15 to 19 years^(3)^.

Suicidal behavior (SB) is perceived as a set of sequential elements of subtle transition, which begins with suicidal ideation, which are thoughts of self-extermination and if there is continuation of the thought, suicidal planning appears, when individuals organize when, where and how they will end their own life, and then a suicide attempt may occur, which may or may not culminate in death^(4)^.

Several studies discuss risk factors for suicide in different contexts and realities, and associations with sociodemographic variables, such as gender, race, social class and marital status, were discussed and identified in some of these works^(5,6)^.

The academic community has been the object of study within this topic. This includes students and other leading actors of this space, such as educators^(7,8,9,10)^.

Bringing the subject into the university is a challenge, but it encourages everyone’s concern, from identifying risk behavior and illness, to strengthening the bond with the mental health service on and off campus^(11)^.

It is noteworthy that the data collection for the present study began months after the second wave of coronavirus in the country, at an atypical moment, with teaching starting from remote to hybrid, and it is known that situational crises reflect impacts on the population’s mental health and worse perceived well- being^(12)^.

Addressing the issue of suicide without alarmism and facing stigmas, encouraging its prevention, can contribute to improving our current situation. Critical, well-founded interventions, based on evidence and reliable data, can be performed in certain groups and with individuals to prevent suicide attempts and avoid death from this cause^(3)^.

Thus, this research aims to determine the risk factors for SB among students and employees of a federal public university in the Western Brazilian Amazon.

## OBJECTIVE

To determine the risk factors for SB among students and employees of a federal public university in the Brazilian Western Amazon.

## METHODS

### Ethical aspects

The research project was submitted to the Research Ethics Committee of the *Universidade Federal do Acre* (UFAC), being approved on April 23, 2021.

### Study design, period and place

This is an analytical cross-sectional study of survey and association between variables, carried out from June 24, 2021 to August 2022, at the *Floresta* campus, UFAC, in Cruzeiro do Sul, Acre, Brazil.

The present study followed the guidelines proposed by STrengthening the Reporting of OBservational studies in Epidemiology (STROBE)^(13)^.

### Population or sample; inclusion and exclusion criteria

The sample consisted of professors, administrative technicians and students from UFAC – *Floresta* campus.

Currently, the *Floresta* campus brings together an estimated population of 1,530 students, arranged in a total of 10 courses, divided into two centers, namely: Center for Education and Languages and Linguistics (CEL), linked to courses in languages and linguistics-English, languages and linguistics-Spanish, pedagogy, languages and linguistics-Portuguese; and the Multidisciplinary Center (MULTIC), which comprises bachelor’s degree biology, bachelor’s degree nursing, agronomy, forest engineering, biology and law courses. The indigenous degree course is offered in a modular format, training the second class in the first half of 2022. All courses participated in the survey. There are, in this institution, 128 professors and 62 active technicians.

After calculating the sample for the population of interest, a minimum of 308 academics, 97 professors and 54 administrative technicians were obtained.

All those who agreed to participate were included, only people under the age of 18 were excluded. Thus, 475 individuals were interviewed, including 97 professors, 54 administrative technicians, 322 students and two interviewees who were both students and administrative technicians.

### Data collection instrument

The following instruments were used:

A – Identification form: contains information about age, gender, sexual orientation, race/skin color, marital status, course and academic period (for students), religion, family income, children, number of residents in the residence, presence of disability and type, use of the Psychosocial Care Center (CAPS - *Centro de Atenção Psicossocial*) of the municipality or Psychological Support Service (PSS) of the university for reasons other than suicidal intent, SB, seeking health services for suicidal reasons, conduct and satisfaction with the care provided.

B – Columbia-Suicide Severity Rating Scale (C-SSRS): to assess suicide risk, the C-SSRS, Portuguese version, was used. Based on study characteristics or clinical need, one of the versions of this scale was chosen, which assesses signs of SB in different periods. In this study, the version called “baseline/screening version” was used, which measures the worst period of suicidal ideation during life and in the last month.

The C-SSRS is divided into four subscales: a) suicidal ideation; b) intensity of ideation; c) SB; d) lethality of effective attempts. The cut-off point is considered when obtaining at least one positive response in ideation, behavior and trial sessions. The necessary training for using the scale was carried out, and the Portuguese version was made available by the authors for use in the present study^(14)^.

### Data collection

Data collection started on June 24, 2021, in a period of remote activities, and happened through contact with each course secretariat to obtain the nominal list, telephone number and email of academics. Contact with public servants took place through the list offered by MULTIC and CEL, with the telephone number and email of each public servant linked to them. The other public servants, who were not linked to any of the centers, but worked effectively in other sectors, such as the sub-prefecture, inclusion support center, for instance, were contacted in the physical space of the campus during the return of on-site activities.

Invitations were sent via WhatsApp® to all contacts provided. As soon as the guest answered, the day and time of their choice were scheduled and, at the agreed time, a link was sent via Google Meet and, from there, the question form link via Google Forms.

After explaining the questionnaires, guaranteeing secrecy, and consenting to the Informed Consent Form (ICF) (online in remote interviews or in writing, in on-site interviews), participants answered alone questions that comprised the identification form, and, in the C-SSRS questions, the questions were asked by the interviewer, and participants checked normally (checking the option that represented them), since the instrument is not self-administered. At the end, a copy of the ICF was sent to participants.

In the virtual or physical environment, if any participant showed signs of depression or deep sadness, crying and/or lack of emotional control, it was suggested that they participate in the service provided by PSS at UFAC, and/or CAPS, according to their preference, since, before the beginning of the research, the professionals of the two services were informed about the nature and purpose of the study, manifesting documental agreement with the possible referral of students or professionals to specialized care.

Data collection was carried out by a single researcher, and was completed in August 2022.

### Analysis of results, and statistics

In the analysis of results, the Statistical Package for the Social Sciences (SPSS) version 23.0 program was used. For continuous variables, mean, standard deviation, median, minimum and maximum were calculated. For categorical variables, frequency and percentage were calculated.

When comparing age with lifetime suicidal ideation (LSI), last month suicidal ideation (LMSI), lifetime suicide planning (LSP) and lifetime suicide attempt (LSA), the Mann-Whitney test was used. To compare categorical variables with LSI, LMSI, LSP and LSA, the chi-square test was used and, when necessary, Fisher’s exact test or the likelihood ratio test. When verifying the factors that best explain LSI, LMSI, LSP and LSA, a logistic regression model (simple and multiple) was used. The selection method used was Forward. A significance level of 5% was used (p value < 0.05).

## RESULTS

A total of 97 (20.4%) professors, 54 (11.4%) administrative technicians, 322 (67.8%) students and two people (0.4%) who were both administrative technicians and students were interviewed. Thus, the final sample consisted of 475 participants, with a mean age of 29.08 (±10.06). Of these, 261 (54.9%) were female; 401 (84.8%) were heterosexual; 26 (5.5%) were homosexual; 305 (64.2%) self-declared as brown; 223 (47%) were single (not even dating at the time of the interview); 279 (61.5%) were religious and practicing, 177 (43.9%), Catholics, 164 (40.7%), Evangelicals; and the rest were from other belief systems.

Of the respondents, 80 (16.8%) were not linked to any specific course (professors of basic subjects, technical-administrative sectors that meet the institution’s general demand, for instance) and 109 (22.9%) were linked to nursing course, which had the highest number of respondents.

The prevalence of LSI was 38.3% and LMSI was 8.3%. Of those who had suicidal ideation, 48.6% performed LSP and 7.6% planned in the last month. Overall, the prevalence of LSA was 10.1% and 0.9% in the last month.

A higher proportion of LSI was found in participants who were younger, were female, were dating/engaged, had no religion, had a monthly family income of less than two minimum wages and/or had no children. Heterosexuals had a lower proportion of LSI compared to homosexual, bisexual and pansexual/other participants. Divorced or widowed participants had a higher proportion of LSI than married/stable union participants. Those with non-practicing religion had a higher LSI proportion than participants with practicing religion (Table 1).

**Table 1:** Sociodemographic characteristics in relation to suicidal ideation at any time in students’, professors’ and administrative technicians’ lives at the *Universidade Federal do Acre* (*Floresta* campus) - June 2021 to August 2022

	Have you ever seriously thought about killing yourself (with no idea about how to kill yourself/associated methods, intentions or plans) (at any time during your life)?		Total	p value
	Yes	No		
Age				
Mean (SD)	26.18 (8.31)	30.96 (10.65)	29.13 (10.08)	<0.0001*
Median	23	28	25	
Minimum-Maximum	17-55	18-60	17-60	
Total number of participants	181	290	471	
Gender				
Female	111 (42.9%)	148 (57.1%)	259 (100%)	0.0263**
Male	70 (32.9%)	143 (67.1%)	213 (100%)	
Total number of participants	181 (38.3%)	291 (61.7%)	472 (100%)	
Sexual orientation				
Heterosexual	133 (33.4%)	265 (66.6%)	398 (100%)	<0.0001**
Homosexual	17 (65.4%)	9 (34.6%)	26 (100%)	
Bisexual	24 (70.6%)	10 (29.4%)	34 (100%)	
Pansexual/other	7 (58.3%)	5 (41.7%)	12 (100%)	
Total number of participants	181 (38.5%)	289 (61.5%)	470 (100%)	
Race/ethnicity				
White	31 (33.7%)	61 (66.3%)	92 (100%)	0.1764**
Brown	118 (38.9%)	185 (61.1%)	303 (100%)	
Black	28 (47.5%)	31 (52.5%)	59 (100%)	
Yellow/indigenous	4 (22.2%)	14 (77.8%)	18 (100%)	
Total number of participants	181 (38.3%)	291 (61.7%)	472 (100%)	
Marital status				
Married/stable union	37 (24.3%)	115 (75.7%)	152 (100%)	<0.0001**
Single	95 (42.6%)	128 (57.4%)	223 (100%)	
Dating/engaged	41 (55.4%)	33 (44.6%)	74 (100%)	
Divorced/widowed	8 (36.4%)	14 (63.6%)	22 (100%)	
Total number of participants	181 (38.4%)	290 (61.6%)	471 (100%)	
Religion				
Yes, practicing	84 (30.3%)	193 (69.7%)	277 (100%)	<0.0001**
Yes, non-practicing	50 (47.6%)	55 (52.4%)	105 (100%)	
No	44 (63.8%)	25 (36.2%)	69 (100%)	
Total number of participants	178 (39.5%)	273 (60.5%)	451 (100%)	
Family income				
Less than 2 wages	85 (52.1%)	78 (47.9%)	163 (100%)	<0.0001**
From 2 to 4 wages	49 (36.3%)	86 (63.7%)	135 (100%)	
From 5 to 10 wages	36 (28.3%)	91 (71.7%)	127 (100%)	
More than 10 wages	11 (23.4%)	36 (76.6%)	47 (100%)	
Total number of participants	181 (38.3%)	291 (61.7%)	472 (100%)	
Do you have children?				
Yes	36 (23.4%)	118 (76.6%)	154 (100%)	<0.0001**
No	145 (45.6%)	173 (54.4%)	318 (100%)	
Total number of participants	181 (38.3%)	291 (61.7%)	472 (100%)	
Presence of physical disability				
Yes	6 (35.3%)	11 (64.7%)	17 (100%)	0.7920**
No	175 (38.5%)	280 (61.5%)	455 (100%)	
Total number of participants	181 (38.3%)	291 (61.7%)	472 (100%)	

*Mann-Whitney test/**Chi-square test. Note: not all participants answered the questions.

Of the respondents who had LMSI, most were younger, female, had no religion or had, but were non-practicing and had no children.

Heterosexual participants had a lower proportion of LMSI than homosexual, bisexual and pansexual/other participants (Table 2).

**Table 2:** Sociodemographic characteristics in relation to suicidal ideation in the last month of students, professors and administrative technicians at the *Universidade Federal do Acre* (*Floresta* campus) - June 2021 to August 2022

	Have you ever really thought about killing yourself (no idea about how to kill yourself/associated methods, intentions or plans) (within the last month)?		Total	p value
	Yes	No		
Age				
Mean (SD)	24.62 (6.11)	29.58 (10.28)	29.16 (10.08)	0.0066*
Median	23	25	25	
Minimum-Maximum	18-40	17-60	17-60	
Total number of participants	39	430	469	
Gender				
Female	30 (11.6%)	228 (88.4%)	258 (100%)	0.0039**
Male	9 (4.2%)	203 (95.8%)	212 (100%)	
Total number of participants	39 (8.3%)	431 (91.7%)	470 (100%)	
Sexual orientation				
Heterosexual	21 (5.3%)	376 (94.7%)	397 (100%)	<0.0001***
Homosexual	5 (19.2%)	21 (80.8%)	26 (100%)	
Bisexual	9 (26.5%)	25 (73.5%)	34 (100%)	
Pansexual/other	4 (36.4%)	7 (63.6%)	11 (100%)	
Total number of participants	39 (8.3%)	429 (91.7%)	468 (100%)	
Race/ethnicity				
White	7 (7.6%)	85 (92.4%)	92 (100%)	0.5195***
Brown	28 (9.3%)	273 (90.7%)	301 (100%)	
Black	4 (6.8%)	55 (93.2%)	59 (100%)	
Yellow/indigenous	0 (0%)	18 (100%)	18 (100%)	
Total number of participants	39 (8.3%)	431 (91.7%)	470 (100%)	
Marital status				
Married/stable union	5 (3.3%)	147 (96.7%)	152 (100%)	0.0513**
Single	23 (10.4%)	198 (89.6%)	221 (100%)	
Dating/engaged	9 (12.2%)	65 (87.8%)	74 (100%)	
Divorced/widowed	2 (9.1%)	20 (90.9%)	22 (100%)	
Total number of participants	39 (8.3%)	430 (91.7%)	469 (100%)	
Religion				
Yes, practicing	10 (3.6%)	266 (96.4%)	276 (100%)	<0.0001**
Yes, non-practicing	14 (13.5%)	90 (86.5%)	104 (100%)	
No	15 (21.7%)	54 (78.3%)	69 (100%)	
Total number of participants	39 (8.7%)	410 (91.3%)	449 (100%)	
Family income				
Less than 2 wages	17 (10.5%)	145 (89.5%)	162 (100%)	0.1180**
From 2 to 4 wages	13 (9.7%)	121 (90.3%)	134 (100%)	
From 5 to 10 wages	9 (7.1%)	118 (92.9%)	127 (100%)	
More than 10 wages	0 (0%)	47 (100%)	47 (100%)	
Total number of participants	39 (8.3%)	431 (91.7%)	470 (100%)	
Do you have children?				
Yes	5 (3.2%)	149 (96.8%)	154 (100%)	0.0056**
No	34 (10.8%)	282 (89.2%)	316 (100%)	
Total number of participants	39 (8.3%)	431 (91.7%)	470 (100%)	
Presence of physical disability				
Yes	0 (0%)	17 (100%)	17 (100%)	0.2065****
No	39 (8.6%)	414 (91.4%)	453 (100%)	
Total number of participants	39 (8.3%)	431 (91.7%)	470 (100%)	

*Chi-square test/***Likelihood ratio test/****Fisher’s exact test. Note: not all participants answered the questions.

A higher proportion of LSP was found in younger participants, respondents who had no religion or had one, but were not practicing.

Heterosexual participants had a lower LSP proportion than homosexual, bisexual and pansexual/other participants, and married/stable union participants had a lower proportion than single, dating/engaged and divorced or widowed participants (Table 3).

**Table 3:** Sociodemographic characteristics in relation to suicidal planning at any time in students’, professors’ and administrative technicians’ lives at the *Universidade Federal do Acre* (Floresta campus) - June 2021 to August 2022

	Have you thought about how you could do this (at any point in your life)?		Total	p value
	Yes	No		
Age				
Mean (SD)	25.67 (7.95)	27.94 (8.88)	26.84 (8.5)	0.0399*
Median	23	24	23	
Minimum-Maximum	17-55	18-54	17-55	
Total number of participants	138	147	285	
Gender				
Female	84 (48.6%)	89 (51.4%)	173 (100%)	0.9845**
Male	55 (48.7%)	58 (51.3%)	113 (100%)	
Total number of participants	139 (48.6%)	147 (51.4%)	286 (100%)	
Sexual orientation				
Heterosexual	97 (42.4%)	132 (57.6%)	229 (100%)	0.0003***
Homosexual	15 (68.2%)	7 (31.8%)	22 (100%)	
Bisexual	21 (80.8%)	5 (19.2%)	26 (100%)	
Pansexual/other	6 (66.7%)	3 (33.3%)	9 (100%)	
Total number of participants	139 (48.6%)	147 (51.4%)	286 (100%)	
Race/ethnicity				
White	25 (48.1%)	27 (51.9%)	52 (100%)	0.2337***
Brown	86 (46.5%)	99 (53.5%)	185 (100%)	
Black	25 (62.5%)	15 (37.5%)	40 (100%)	
Yellow/indigenous	3 (33.3%)	6 (66.7%)	9 (100%)	
Total number of participants	139 (48.6%)	147 (51.4%)	286 (100%)	
Marital status				
Married/stable union	24 (33.8%)	47 (66.2%)	71 (100%)	0.0370**
Single	76 (52.8%)	68 (47.2%)	144 (100%)	
Dating/engaged	34 (55.7%)	27 (44.3%)	61 (100%)	
Divorced/widowed	5 (50%)	5 (50%)	10 (100%)	
Total number of participants	139 (48.6%)	147 (51.4%)	286 (100%)	
Religion				
Yes, practicing	61 (38.9%)	96 (61.1%)	157 (100%)	0.0002**
Yes, non-practicing	40 (54.1%)	34 (45.9%)	74 (100%)	
No	37 (71.2%)	15 (28.8%)	52 (100%)	
Total number of participants	138 (48.8%)	145 (51.2%)	283 (100%)	
Family income				
Less than 2 wages	65 (56%)	51 (44%)	116 (100%)	0.1360**
From 2 to 4 wages	41 (47.7%)	45 (52.3%)	86 (100%)	
From 5 to 10 wages	24 (38.7%)	38 (61.3%)	62 (100%)	
More than 10 wages	9 (40.9%)	13 (59.1%)	22 (100%)	
Total number of participants	139 (48.6%)	147 (51.4%)	286 (100%)	
Do you have children?				
Yes	28 (40.6%)	41 (59.4%)	69 (100%)	0.1259**
No	111 (51.2%)	106 (48.8%)	217 (100%)	
Total number of participants	139 (48.6%)	147 (51.4%)	286 (100%)	
Presence of physical disability				
Yes	4 (40%)	6 (60%)	10 (100%)	0.5796****
No	135 (48.9%)	141 (51.1%)	276 (100%)	
Total number of participants	139 (48.6%)	147 (51.4%)	286 (100%)	

*Chi-square test/***Likelihood ratio test/****Fisher’s exact test. Note: not all participants answered the questions.

Respondents who attempted suicide during their lives were mostly younger, female, had no religion, and had lower monthly family income. Heterosexual participant presents or lower proportion of LSA than participant of other sexual orientations (homosexual, bisexual and pansexual/other). Married/stable union participants had a lower proportion of LSA than single, dating/engaged and divorced or widowed participants (Table 4).

**Table 4:** Sociodemographic characteristics in relation to suicide attempts at any time in students’, professors’ and administrative technicians’ lives at the *Universidade Federal do Acre* (Floresta campus) - June 2021 to August 2022

	Have you made a suicide attempt (at any time during your life)?		Total	p value
	Yes	No		
Age				
Mean (SD)	25.27 (7.92)	29.47 (10.17)	29.04 (10.04)	0.0023*
Median	22	25	25	
Minimum-Maximum	17-55	18-60	17-60	
Total number of participants	48	425	473	
Gender				
Female	33 (12.6%)	228 (87.4%)	261 (100%)	0.0443**
Male	15 (7%)	198 (93%)	213 (100%)	
Total number of participants	48 (10.1%)	426 (89.9%)	474 (100%)	
Sexual orientation				
Heterosexual	31 (7.8%)	369 (92.3%)	400 (100%)	0.0005***
Homosexual	5 (19.2%)	21 (80.8%)	26 (100%)	
Bisexual	9 (26.5%)	25 (73.5%)	34 (100%)	
Pansexual/other	3 (25%)	9 (75%)	12 (100%)	
Total number of participants	48 (10.2%)	424 (89.8%)	472 (100%)	
Race/ethnicity				
White	10 (10.8%)	83 (89.2%)	93 (100%)	0.9929**
Brown	30 (9.8%)	275 (90.2%)	305 (100%)	
Black	6 (10.3%)	52 (89.7%)	58 (100%)	
Yellow/indigenous	2 (11.1%)	16 (88.9%)	18 (100%)	
Total number of participants	48 (10.1%)	426 (89.9%)	474 (100%)	
Marital status				
Married/stable union	7 (4.6%)	145 (95.4%)	152 (100%)	0.0302**
Single	31 (13.9%)	192 (86.1%)	223 (100%)	
Dating/engaged	7 (9.2%)	69 (90.8%)	76 (100%)	
Divorced/widowed	3 (13.6%)	19 (86.4%)	22 (100%)	
Total number of participants	48 (10.1%)	425 (89.9%)	473 (100%)	
Religion				
Yes, practicing	22 (7.9%)	256 (92.1%)	278 (100%)	<0.0001**
Yes, non-practicing	7 (6.6%)	99 (93.4%)	106 (100%)	
No	19 (27.5%)	50 (72.5%)	69 (100%)	
Total number of participants	48 (10.6%)	405 (89.4%)	453 (100%)	
Family income				
Less than 2 wages	25 (15.3%)	138 (84.7%)	163 (100%)	0.0122**
From 2 to 4 wages	15 (11%)	121 (89%)	136 (100%)	
From 5 to 10 wages	6 (4.7%)	123 (95.3%)	129 (100%)	
More than 10 wages	2 (4.3%)	44 (95.7%)	46 (100%)	
Total number of participants	48 (10.1%)	426 (89.9%)	474 (100%)	
Do you have children?				
Yes	10 (6.5%)	144 (93.5%)	154 (100%)	0.0689**
No	38 (11.9%)	282 (88.1%)	320 (100%)	
Total number of participants	48 (10.1%)	426 (89.9%)	474 (100%)	
Physical Disability				
Yes	1 (5.9%)	16 (94.1%)	17 (100%)	0.5547****
No	47 (10.3%)	410 (89.7%)	457 (100%)	
Total number of participants	48 (10.1%)	426 (89.9%)	474 (100%)	

*Chi-square test/***Likelihood ratio test/****Fisher’s exact test. Note: not all participants answered the questions.

In the multiple logistic regression, the variables that best explained LSI were gender, sexual orientation, religion, family income and having children. The variables that best explained LMSI were gender, sexual orientation and religion. The variables that best explained LSP were sexual orientation and religion. LSA was best explained by variables gender, sexual orientation, marital status and religion.

## DISCUSSION

Suicidal ideation is an almost essential component of the process called SB, appearing as a stimulus associated with the other elements^(15)^.

Veloso *et al.,* in a study entitled “*Ideação suicida em universitários da área da saúde: prevalência e fatores associados*”, emphasize the importance of attempts and redirect to research showing that a history of suicide attempts has predictive value in assessing suicide risk^(7)^.

As identified in other epidemiological studies, SB was more prevalent in youth, among females, singles and with low family income, demonstrating greater fragility in this group, especially^(12)^.

There is a segment of factors related to SB among young people. Among them are sadness, hopelessness, depression, anxiety, low self-esteem, previous traumatic experiences such as physical and sexual abuse, few friendships and little emotional support, rejection and substance use. Adolescence and early adulthood are indicated as the main stages of life in which this set of behaviors can precipitate SB^(3)^.

Generation Y, which is called the “millennial generation”, encompassing those born between 1981 and 1995, and generation Z, which are the “digital born”, those born after 1995, carry particularities that affect the growth of SB in young adults, according to the literature. Generation Z, for example, realized that they are more sensitive to stress, and therefore manifest more anxiety, depression, self-mutilation and SB. Less resilience and immediacy was identified in them^(3)^.

Special mention should be made of the iGen generation, born between 1995 and 2012, who grew up with early contact with the internet and with technologies to connect to it, which has raised concerns about exposure to cyberbullying, access to harmful or damaging materials and information, less tangibility of social relationships, slower pace of maturation towards adulthood and less religiosity. More than that, the iGen generation is also marked by a higher incidence of mental disorders^(5)^.

In this study, females had a higher proportion of LSI and LMSI and LSA than males. Several authors highlight the difference between the sexes as a significant factor in suicide risk, since, worldwide, males have a higher death risk by suicide than females. In the latter, there is a higher prevalence of ideation and attempts. However, men, by employing more lethal means and by having greater access to suitable objects and even greater aggressiveness, make lethal attempts^(1,2,3,4,5)^.

Santos *et al*. found a 9.9% LMSI prevalence in a study carried out with 637 students at a federal university in Mato Grosso. Lower income, non-heterosexual orientation and not having a religious practice were associated with LMSI^(16)^.

Not being heterosexual in the present study was proportionally related to LSI, LMSI, LSP and LSA. Having a sexual orientation that is not the “socially expected” one can have different consequences among individuals who define and assume, and may be the target of prejudice, generating immense suffering as well as intense emotional fragility, leading to SB production^(16)^.

Regarding marital status, participants who were married or in a stable relationship had lower proportions in the different SB categories. Such findings are in line with a study by Aguiar *et al*., who found, in a study carried out with individuals aged 18 or over assisted in the urban Primary Health Care network in Passo Fundo, Rio Grande do Sul, prevalence of suicide attempt of 9% and association with the absence of a spouse, in addition to adulthood, being female, lower education, diagnosis of chronic diseases, insomnia and family history of suicide^(17)^.

Suicide risk was flagged in those living alone, with the highest rates among those who were divorced or never married^(5)^.

Not having a religion and/or having and being non-practicing were related to SB. The exercise of religious practice such as praying, meditating and belief conducts contributes to balancing emotions and feelings. Thus, having a religious practice can manifest itself as a protective factor for individuals regarding the onset of SB^(5,16)^.

As for income, it is difficult to find studies that directly relate suicide to poverty, but there are indications that, in historical moments of economic crisis, as in the context in which data collection was carried out, suicide numbers increase significantly. Social humiliation leads to subjective impacts within this asymmetry, helping to understand the dimension of suffering linked to inequality, financial degradation and, at certain times, the moral degradation of some groups or people in certain circumstances with ideas such as “no power” and “not being able to”^(5)^.

Even though the sample of our study is composed in a larger number of students, proportionally, those without children had a higher percentage of SB.

In research with a population of 240 individuals aged between 18 and 68 years, equally divided into control and experimental groups, most who attempted suicide said they did not have children, and the number of attempts in those who had experienced this practice was inversely proportional to the number of children^(18)^.

This finding, as the authors point out, is in line with the literature, which highlights the presence of children at home as one of the main protective factors for SB, or the existence of children^(5)^.

In 2021, a study was carried out based on care provided in the psychiatric emergency to 130 individuals with ideation, planning or suicide attempt, which aimed to analyze the clinical aspects and factors associated with SB in the COVID-19 pandemic, using notes on sociodemographic, clinical and therapeutic characteristics, and also the identification of care needs.

In that study, SB was revealed by suicide attempt, ideation and planning, mainly in females, in young adults, unemployed and with low family income as well as in patients who had reports of mental disorder, psychiatric hospitalization, previous attempts to suicide and abandonment. Loss of income and previous hospitalization were associated with SB manifestation^(12)^.

The aforementioned work is contemporary with the present study, where it should be noted that the moment of the COVID-19 pandemic brought, as some of its consequences, mental illness and hopeless thoughts, which may contribute in some way to the results found. However, as there are no previous measurements of SB in this institution or even in the city, we can only say that the results discussed here are in line with those described in other institutions and locations in the country, and it is not possible to say how much the academic community was affected by this anomalous moment.

It is important to emphasize suicide as a complex and multifaceted phenomenon, with a multifactorial etiology, which cannot be fully explained and understood from a single focus, since, in this way, the analysis would be partial and fragmented^(5)^.

SB involves from distal factors, which include traumatic experiences at the beginning of life, in addition to psychoactive genetic characteristics, to proximal factors, which are adverse experiences throughout life and abuse of alcohol and other drugs in a deleterious way, and it is necessary to view suicide as an individual experience, permeated by the dualism between the will to die, to end suffering, and the yearning for help. Furthermore, it is noteworthy that 80% of SB cases are associated with the presence of a mental disorder, according to the literature^(3)^.

For the present time, movements such as the one proposed by the psychology course at the *Universidade Federal do Tocantins*, which, in an extension action entitled “Surviving Suicide”, intended to foster dialogue about mental health. The project was based on cycles of debates, discussing suicide as a public health and mental health issue, and had the academic community and the community in general as target audiences. In conversation circles, an attempt was made to welcome participants and clarify doubts regarding the topic of suicide^(11)^.

### Study limitations

Although we generalize the academic community within this theme, when we include professors, administrative technicians and students in the same analysis, knowing that there are possibly differences in style and quality of life, personal experiences and ways of facing challenges between the three groups, which can be this is a limitation of this study, we can visualize the campus’ current situation, with a panoramic view, realizing that a work of promoting a mental health culture must be offered holistically.

### Contributions to nursing, health, or public policies

We cannot neglect the discussion about SB, as it causes suffering to those who experience the ambivalence of this experience. Its apogee generates social loss, public spending, emotional pain for victims’ loved ones, who experience grief in search of meanings for the loss with short and/or long-term negative repercussions. Therefore, it is necessary to have theoretical-practical knowledge, reception and proximity, with this relevant and multifaceted theme, for better management and efficiency in the service provided, immersing in the particularities of each group that it is possible to approach.

## CONCLUSIONS

Within the proposal, this study suggests a relationship between sociodemographic factors and SB in the studied academic community. A higher proportion of SB was found in younger participants. The variables that best explained SB were gender, sexual orientation, religion, family income and having children. It reinforces that mental health should be promoted in the academic environment, with the creation of spaces for listening and mutual help, such as conversation circles. It is possible that other types of studies, even longitudinal ones, carried out in Higher Education Institutions in Brazil, provide a better understanding of SB, filling gaps in knowledge identified with the accomplishment of this work.

## Supplementary Material

0034-7167-reben-76-s2-e20230102-suppl01

## Data Availability

https://doi.org/10.48331/scielodata.PHPQLK
