# Supplementary material for: Sociodemographic factors associated with suicidal behavior at a federal public university in the Western Brazilian Amazon
Source: Rev Bras Enferm. 2023 Dec 8;76(Suppl 2):e20230102. doi: 10.1590/0034-7167-2023-0102 (PMC10704696; doi:10.1590/0034-7167-2023-0102)
Supplement: 0034-7167-reben-76-s2-e20230102-suppl01 [file 0034-7167-reben-76-s2-e20230102-suppl01.pdf]

Genero OrientacaoSexual Idade Raca_Cor EstadoCivil Curso Ocupacao
Periodo_Academico Religiao Qual_religiao Renda_Familiar Filhos
Quantos_Filhos Pessoas_na_casa Deficiencia_Fisica Uso_do_SAP_NCS
Uso_do_CAPS_NCS Teve_CS Buscou_ajuda Qual Conduta
Satisfeito Como_avaliaria_sua_qualidadedevida Satisfacao_com_a_saude
Em_que_medida_suador_fisica_impede_de_fazer_o_que_precisa
Tratamento_medico_para_levar_sua_vida Aproveita_a_vida Sentido_da_vida
Consegue_se_concentrar Segurança Saude_do_ambiente_fisico Energia
Aceitacao_da_aparencia_fisica Dinheiro_para_necessidades
Informacoes_diarias Oportunidade_de_lazer Locomocao
Satisfacao_com_o_sono Capacidade_de_desempenhar_as_atividades
Capacidade_para_o_trabalho Satisfacao_consigo
Satisfacao_com_relacoes_pessoais Satisfacao_sexual Apoio_dos_amigos
Local_em_que_mora Acesso_servicos_de_saude Meio_de_Transporte
Ambiente_academico Sentimentos_negativos
Desejou_estar_morto_dormir_e_4_mais_acordar_na_vida
Desejou_estar_morto_dormir_e_4_mais_acordar_no_ultimo_mes
Pensou_em_se_matar_sem_planos_na_vida
Pensou_em_se_matar_sem_planos_no_ultimo_mes
Pensado_em_como_poderia_fazer_isso_na_vida
Pensado_em_como_poderia_fazer_isso_no_ultimo_mes
Intencao_de_colocar_em_pratica_na_vida
Intencao_de_colocar_em_pratica_no_ultimo_mes
Detalhes_de_como_se_matar_e_execucao_na_vida
Detalhes_de_como_se_matar_e_execucao_no_ultimo_mes
Numero_de_vezes_navida Numero_de_vezes_no_ultimo_mes
Quanto_tempo_eles_duravam_na_vida Quanto_tempo_eles_duram_no_ultimo_mes
Conseguiu_parar_de_pensar_em_se_matar_ou_querer_morrer_navida
Conseguiu_parar_de_pensar_em_se_matar_ou_querer_morrer_no_ultimo
Coisas_que_impediram_de_querer_morrer_ou_suicidio_na_vida
Coisas_que_impediram_de_querer_morrer_ou_suicidio_no_ultimo_mes
Razao_qualquer_momento_da_vida Razao_ultimo_mes Tentativa_na_vida
NTentativas_na_vida Tentativa_ultimos_3_meses
Ntentativas_ultimos_3_meses Comportamento_autolesivo_na_vida
Comportamento_autolesivo_ultimos_3_meses Comecou_e_alguem_impediu_na_vida
Ntentativas_interrompidas_na_vida Comecou_e_alguem_impediu_ultimo_3meses
Ntentativas_interrompidas_nosultimos_3meses
Comecou_tentativa_e_parou_navida Ntentativas_abortadas_na_vida
Comecou_tentativa_e_parou_ultimos_3meses
NTentativasabortadas_ultimos_3meses Deu_algum_passo_na_vida
Deu_algum_passo_nos_ultimos_3meses Tentativa_mais_recente
Letalidade_efetiva_Danosfisicos_recente Letalidade_potencial_recente
Tentativa_mais_letal Letalidade_efetiva_Danosfisicos_letal
Letalidade_potencial_letal Tentativa_inicial_primeira
Letalidade_efetiva_Danosfisicos_primeira Letalidade_potencial_primeira

1 1 34 2 1 1 1 11 1 1 4 1 1 2
2 1 2 2 2 1 2 2 2 4
5 3 3 4 3 4 3 5 4 2 2 4 3
2 2 2 2 1 3 1 3 2 2 2 2 2

2 2 2 2 2 2
2 2 2 2

1 1 32 1 1 2 2 11 1 1 4 1 2 4
2 2 2 2 3 2 2 1 2 3
4 3 3 4 3 4 3 4 3 1 1 2 3
2 2 3 2 2 2 2 3 2 2 2 2 2

2 2 1 1 2 2
2 2 2 2

1 1 44 1 2 1 1 11 1 1 3 2 2
2 2 1 2 3 2 4 4 3 4
4 3 3 4 3 3 4 4 2 1 4 2 3

	2	2	2	2	2	2	2	2	2	1	2	2	2
	2	2	2	2	2	2	3		4		3		1
		4		2		2		1	2	2		2	
	2		2		1	2							
1	1	32	2	1	12	2	11	1	1	3	2		2
	2	2	2	2	3				2	2	1	1	4
	4	4	3	5	4	4	3	4	4	2	1	2	2
	2	2	1	2	1	2	1	3	3	2	2	2	2
				2		2		2	2	2		2	
	2		2		2	2							
1	1	52	2	2	1	2	11	1	1	3	2		6
	2	2	2	1	1	6	5	1	2	2	2	2	4
	4	3	4	4	4	5	4	4	3	2	4	2	2
	2	1	3	2	2	2	1	2	3	1	2	1	2
	1	2	1	2	1	2	5		1		2		1
		3		2		2		2	2	2		2	
	2		2		2	2							
2	1	42	2	1	12	2	11	1	4	3	1	1	2
	2	1	2	2	3				2	2	1	1	4
	5	4	4	4	3	4	3	4	3	1	2	2	2
	2	2	2	2	2	3	2	2	3	2	2	2	2
				2		2		2	2	2		2	
	2		2		2	2							
2	1	44	1	1	2	1	11	1	1	3	1	1	3
	2	2	2	2	3				1	2	2	2	4
	4	3	4	4	4	5	4	4	3	2	1	2	2
	2	2	2	3	2	2	2	2	3	2	2	2	2
				2		2		2	2	2		2	
	2		2		2	2							
1	1	38	1	1	2	2	11	1	2	2	1	1	3
	2	2	1	2	3				2	2	1	1	4
	5	4	4	4	3	4	4	4	4	2	3	2	2
	2	2	2	2	1	4	4	2	3	1	2	2	2
	2	2	2	2	2	2	1		2		1		6
		6	6	2		2		2	2	2		2	
	2		2		1	2	35891	1	2	35891	1	2	
	35891	1	2										
2	1	42	2	4	6	4	8	1	1	3	2		2
	2	2	2	2					2	2	1	1	3
	5	4	4	4	3	5	3	4	3	2	3	2	1
	2	1	2	1	2	3	2	4	4	2	2	2	2
				2		2		2	2	2		2	
	2		2		2	2							
1	1	27	2	4	12	2	11	1	4	2	2		1
	2	2	2	1	1	3	4	2	2	2	4	3	5
	5	3	4	4	3	4	4	3	3	2	1	2	2
	2	3	1	3	2	2	2	2	3	1	2	1	2
	1	2	1	2	1	2	3		2		3		6
		6		1	5	2		1	2	1	4	2	
	1	2	2		2	2	40306	1	1	40308	1	2	
	40305	1	1										
1	1	36	1	1	1	1	11	1	1	3	1	1	3
	2	2	2	2	3				2	2	2	2	4
	5	3	3	4	4	3	3	3	4	2	2	2	2

	2	2	2	3	2	4	1	2	2	2	2	2	2
				2		2		2	2	2		2	
	2		2		2	2							
1	1	33	2	1	12	2	11	1	2	2	1	3	5
	2	2	2	2	3				2	2	3	2	3
	4	4	4	3	4	4	3	4	3	1	3	2	2
		3	2	3	2	3	2	2	2	1	2	2	2
	2	2	2	2	2	2	1		1		1		1
		5		2		2		2	2	2		2	
	2		2		2	2							
1	1	34	2	1	1	1	11	1	2	4	1	2	4
	2	2	2	2	3				1	1	1	1	4
	5	4	4	4	4	4	4	4	3	2	2	2	2
	2	2	2	2	2	2	1	2	3	2	2	2	2
				2		2		2	2	2		2	
	2		2		2	2							
2	1	40	2	2	1	2	11	1	1	3	1	2	4
	2	2	2	2	3				2	2	1	2	3
	5	4	4	4	4	4	3	4	3	2	2	2	2
	2	1	2	2	3	5	2	2	3	2	2	2	2
	2	2	2	2	2	2							
				2		2		2	2	2		2	
	2		2		2	2							
1	1	54	2	1	1	1	11	2	1	3	1	3	5
	2	2	2	2					2	3	1	3	2
	4	3	4	4	3	3	3	4	3	1	2	2	2
	2	2	3	2	1	4	2	4	3	1	2	2	2
	2	2	2	2	2	2	1		1		1		6
		6		2		2		2				2	
	2		2		2	2							
1	1	33	2	5	12	2	11	1	1	3	1	1	3
	2	1	2	2	3				3	3	5	4	3
	1	3	1	4	2	5	5	5	2	1	5	5	5
	5	1	3	1	1	1	1	1	5	1	1	1	1
	1	1	1	1	2	2	4	4	2	2	1	1	1
	1	5	5	2		2		2	2	2		2	
	2		2		2	2		3					
1	1	45	2	1	12	1	11	1	1	2	1	2	3
	2	2	2	2	3				2	3	2	3	2
	4	4	3	4	3	4	2	3	3	4	4	4	3
	3	2	3	3	2	4	4	3	1	2	2	2	2
				2				2					
	2		2		2	2							
2	1	49	1	1	12	1	11	2	1	4	2		2
	2	2	2	2	3				2	3	2	2	3
	4	4	4	4	3	5	3	4	3	1	4	3	2
	3	3	4	3	2	3	2	2	3	1	2	1	2
	1	2	2	2	2	2	1		3		1		6
	6	5	6	2	0	2	0	2	2	2	0	2	0
	2	0	2	0	2	2							
2	1	42	1	1	12	1	11	1	4	3	1	3	4
	2	2	2	2	3				2	2	1	2	4
	4	4	4	4	4	4	3	4	4	1	2	2	2

	2	2	2	2	2	2	2	2	3	2	2	2	2
				2	0	2		2	2	2		2	
	2		2		2	2							
2	1	38	2	1	12	2	11	1	2	2	1	1	3
	2	2	1	2	3				1	1	1	1	4
	5	3	5	4	5	5	3	5	3	2	1	1	1
	1	1	1	2	1	3	1	3	3	2	2	2	2
				2		2		2	2	2		2	
	2		2		2	2							
2	1	51	1	1	12	2	11	2	2	3	1	3	2
	2	1	2	2	3				2	2	2	2	4
	3	4	4	4	3	4	3	4	4	1	4	4	3
	4	2	2	2	2	3	2	2	3	1	2	1	2
	2	2	2	2	2	2	1		2		2		1
		4		2	0	2	0	2	2	2		2	0
	2	0	2	0	2	2							
1	3	31	1	2	6	1	11	3		3	2		1
	2	1	2	1	2				2	3	4	4	4
	2	3	4	3	3	3	3	3	3	2	4	4	4
	3	3	4	1	3	3	5	5	1	1	1	1	1
	1	2	1	2	2	2	2	1	2	1	2	2	1
	1	3	4	2		2		2	2	2		2	
	2		2		2	2							
2	2	35	1	3	2	1	11	2	6	3	2		1
	2	2	2	1	2		5	2	3	2	3	4	2
	2	3	3	3	3	3	3	3	2	3	3	3	3
	3	4	2	3	4	5	3	4	1	1	1	1	2
	1	2	1	2	2	2	2		3	1	3		4
		5		2		2		1	2	2		2	
	2		2		1	2							
1	1	35	2	1	12	2	11	2	2	3	1	2	4
	2	2	2	2	3				2	1	1	1	4
	4	4	4	3	4	4	3	4	3	2	3	1	1
	1	2	2	2	2	4	1	2	3	1	2	2	2
	2	2	2	2	2	2	1		1		1		6
		6		2		2		2	2	2		2	
	2		2		2	2							
2	1	37	1	2	12	1	11	1	2	2	1	1	1
	2	2	2	2	3				4	4	2	1	3
	5	3	4	4	3	3	4	4	4	1	3	2	3
	2	2	2	2	4	4	2	2	3	2	2	2	2
				2		2		2	2	2		2	
	2		2		2	2							
2	1	44	2	1	2	1	11	1	1	4	1	2	6
	2	2	2	2	3				1	2	1	1	4
	5	4	4	4	4	4	3	4	3	2	2	1	1
	1	2	2	2	1	2	2	1	3	2	2	2	2
				2		2		2	2	2		2	
	2		2		2	2							
1	1	36	2	1	2	1	11	2	3	4	1	2	4
	2	2	2	2	3				2	2	2	3	4
	5	3	4	4	4	4	4	4	5	2	1	3	3

	2	2	2	1	1	1	1	2	2	2	2	2	2
				2		2		2	2	2		2	
	2		2		2	2							
2	1	49	1	1	4	1	11	2	4	4	1	1	3
	2	2	2	1	2				1	2	1	2	4
	5	5	4	4	4	4	4	4	4	2	2	2	2
	2	2	2	2	2	2	1	1	3	1	2	1	2
	1	2	1	2	1	2	1		1		1		1
		4		2		2		2	2	2		2	
	2		2		2	2							
2	2	39	1	3	12	1	11	3		3	2		2
	2	2	2	1	2				1	2	2	2	4
	4	4	4	5	4	4	4	4	4	2	2	2	2
	2	2	3	2	2	2	1	2	3	1	2	1	2
	1	2	1	2	2	2	1		1		1		3
		4		2		2		1	2	2		2	
	2		2		2	2							
2	2	55	1	2	5	1		1	4	3	2		1
	2	2	2	1	1	3	6	2	2	2	2	2	4
	4	4	4	3	4	5	3	3	3	2	3	2	2
	2	2	4	2	2	4	2	2	3	1	2	1	2
	1	2	1	2	1	2	4		2		5		1
		4		1		2		2	2	1		2	
	1				1	2	43374	1	3	43374	1	3	
	43374	1	3										
1	1	33	2	1	1	1		1	1	3	1	1	3
	2	2	2	1	2				2	3	1	3	3
	3	3	3	4	3	3	3	4	4	2	2	4	4
	4	3	3	4	2	3	2	3	2	1	2	1	1
	1	2	1	2	2	2	2		3		2		1
		4		2		2		2	2	2		2	
	2		2		1	2							
1	1	34	1	2	6	1	11	1	1	4	2		1
	2	2	2	2	2				2	2	2	1	4
	5	4	5	5	4	4	5	4	4	2	4	2	2
	2	2	2	2	1	2	2	2	3	2	2	2	2
				2		2		2	2	2		2	
	2		2		2	2							
2	1	32	2	1	2	2	11	1	1	3	1	1	4
	2	2	2	2	2				1	2	2	2	4
	5	4	4	3	3	5	3	5	4	2	2	2	1
	2	2	1	2	1	2	2	1	3	2	2	2	2
				2		2		2	2	2		2	
	2		2		2	2							
2	1	32	2	1	12	1	11	3		4	2		2
	2	2	2	2	3				2	3	1	1	4
	5	4	4	4	3	3	4	3	3	2	2	2	2
	2	2	2	3	2	3	2	3	3	2	2	2	2
				2		2		2	2	2		2	
	2		2		2	2							
1	1	30	2	1	12	2	11	1	2	3	1	1	4
	2	2	2	1	2				1	2	1	1	4
	4	4	4	4	3	4	4	3	3	1	1	3	2

	2	1	3	1	1	2	1	1	3	1	2	1	2
	2	2	2	2	2	2	1		1		2		1
		4		2		2		2		2		2	
	2				2	2							
2	1	37	2	1	2	1	11	1	1	3	1	2	4
	2	2	2	2	3				1	1	1	1	4
	5	4	4	4	4	5	4	4	3	2	2	2	2
	1	2	3	1	2	1	1	2	3	2	2	2	2
				2		2		2	2	2		2	
	2		2		2	2							
2	1	35	3	3	12	2	11	1	4	2	2		2
	2	2	1	2	3				3	4	3	4	3
	4	3	3	3	3	5	2	4	4	2	5	2	2
	3	1	1	1	3	3	1	4	1	1	2	2	2
	2	2	2	2	2	2	1		1		3		6
		5		2	0	2	0	1	2	2	0	2	0
	2	0	2	0	2	2							
2	1	34	3	1	2	1	11	1	2	3	1	1	3
	2	2	2	2	3				1	1	1	1	4
	4	3	3	5	5	5	5	4	2	2	4	3	3
	2	1	1	1	1	1	1	1	3	2	2	2	2
				2		2		2	2	2		2	
	2		2		2	2							
1	1	41	2	1	2	2	11	2	1	2	1	1	3
	2	2	2	2	3				2	1	2	2	4
	5	4	4	4	3	5	3	3	3	2	1	1	1
	2	2	2	2	1	4	3	2	4	2	2	2	2
				2		2		2	2	2		2	
	2		2		2	2							
2	1	35	3	1	2	1	8	1	1	4	1	2	4
	2	2	2	2	3				1	3	2	2	4
	4	3	4	4	5	5	5	5	3	2	1	2	2
	2	2	1	2	2	3	2	4	4	2	2	2	2
				2		2		2	2	2		2	
	2		2		2	2							
1	1	31	2	1	12	2	11	1	1	4	1	1	2
	2	2	2	2	3				1	1	1	1	4
	4	3	4	4	4	4	3	4	4	1	1	2	2
	2	1	1	1	1	2	2	2	3	2	2	2	2
	2	2	2	2	2	2							
				2		2		2	2	2		2	
	2		2		2	2							
2	1	50	2	1	6	1	11	2	2	4	1	2	4
	2	2	2	2	3				2	3	3	3	3
	4	3	3	3	3	4	3	3	2	2	3	3	2
	2	2	2	3	2	2	2	3	3	2	2	1	2
	2	2	2	2	2	2	1	1	1	1	1	1	1
	1	6	6	2		2		2	2	2		2	
	2		2		2	2	43383	1	2	43383	1	2	
	43383	1	2										
1	1	17	2	2	1	3	2	1	2	1	2		4
	2	2	2	1	2				2	4	2	2	3
	4	4	3	2	3	1	3	3	2	2	3	3	3

	4	3	1	2	3	3	4	3	3	1	2	1	2
	1	2	1	2	1	2	1		1		1		1
		6		1	1	2		2	2	1	1	2	
	2		2		1	2	42887	3	2	42896	3	2	
	42896	3	2										
1	1	21	2	2	1	3	2	1	2	3	2		3
	2	2	2	2	3				2	2	2	2	4
	4	3	3	2	3	2	3	3	3	1	4	3	3
	4	3	2	2	3	2	2	4	2	1	1	2	2
	2	2	2	2	2	2			1		2		6
		4		2		2		2	2	2		2	
	2		2		2	2							
1	1	25	2	2	1	3	6	2	2	1	2		6
	2	2	1	2	2				2	2	2	3	4
	3	3	1	3	3	3	3	4	3	1	5	4	4
	5	3	3	3	3	4	5	5	5	1	1	1	1
	1	1	2	2	2	2	3	3	2	2	1	1	1
	1	4	4	2		2		2	2	2		2	
	2		2		2	2							
1	3	19	2	2	1	3	2	2	1	2	2		2
	2	2	2	2	3				2	2	4	2	3
	4	3	3	4	3	3	3	4	4	2	1	2	2
	2	1	2	1	2	2	3	2	3	2	2	2	2
				2		2		2	2	2		2	
	2		2		2	2							
2	1	20	1	2	1	3	2	1	2	1	2		3
	1	2	2	2	3				1	2	3	5	5
	5	5	4	4	5	3	1	4	5	3	3	2	1
	3	2	1	1	1	4	3	2	1	2	2	2	2
				2		2		1	1	2		2	
	2		2		2	2							
1	3	18	2	3	1	1	2	2	1	2	2		2
	2	2	2	2	3				2	2	4	2	4
	5	4	4	4	3	3	3	3	3	1	4	2	2
	2	2	2	2	2	2	2	2	1	1	2	1	2
	1	2	2	2	2	2	1		1		1		1
		6		2		2		1	1	2		2	
	2		2		2	2							
1	1	26	2	1	12	2	11	1	1	3	2		2
	2	2	2	1	2				1	1	1	1	4
	4	3	4	4	3	4	3	4	4	2	1	1	2
	2	2	2	2	3	2	2	3	3	1	2	1	2
	1	2	1	2	2	2	1		1		2		1
		1		2		2		2	2	2		2	
	2		2		2	2							
1	1	37	2	1	3	1	11	1	2	3	2		2
	2	2	2	2	3				2	2		2	3
	5	4	4	4	4	5	5	4	3	2	1	2	2
	2	2	1	2	2	2	2	2	3	1	2	1	2
	2	2	2	2	2	2	1		1		1		6
		6		2		2		2	2	2		2	
	2		2		2	2							
1	1	46	1	4	12	1	11	2		3	1	1	2
	2	1	1	2	3				2	2	1	4	3
	4	3	4	4	3	4	3	4	2	2	2	2	2

	2	2	2	2	2	4	2	2	3	1	2	2	2
	2	2	2	2	2	2	1		2		3		6
		5		2		2		2	2	2			
	2		2		2	2							
1	1	31	2	1	12	2	11	1	1	4	1	1	2
	2	2	2	2	3				1	1	1	1	4
	4	3	4	4	4	4	3	4	4	1	1	2	2
	2	1	1	1	1	2	2	2	3	2	2	2	2
	2	2	2	2	2	2							
				2		2		2	2	2		2	
	2		2		2	2							
1	1	22	2	2	1	3	4	3		1	2		4
	2	2	2	2	1				3	4	4	3	2
	2	3	2	3	1	3	2	2	2	4	4	4	4
	3	2	3	2	5	5	5	3	2	1	1	1	1
	2	2	2	2	1	1	1	1	3	3	3	3	1
	1	5	4	2		2		2	2	2		2	
	2		2	0	2	2							
1	1	20	2	2	1	3	4	2	2	2	2		3
	2	2	2	2	3				2	2	3	2	4
	3	3	3	3	3	3	3	3	3	2	4	2	3
	2	2	2	2	2	2	2	2	3	1	2	1	2
	2	2	2	2	2	2	1		2		2		1
		3		2		2		2	1	2		2	
	2		2		2	2							
1	1	21	1	3	1	3	2	1	2	1	2		3
	1	2	2	1	2				2	4	3	3	4
	4	3	2	2	3	2	3	3	2	1	4	4	4
	4	2	2	3	2	4	4	3	2	2	2	1	2
	1	2	2	2	2	2	2		1		1		1
		5		2		2		2		2		2	
	1	1	2		2								
2	1	30	2	3	6	1	1	3		3	2		3
	2	2	2	2	3				2	3	2	2	3
	1	4	3	3	4	4	3	4	3	1	3	3	2
	3	1	2	2	2	3	2	4	3	2	2	2	2
				2		2		1	2	2		2	
	2		2		2	2							
1	1	19	2	2	1	3	2	1	1	2	2		6
	2	2	2	2	3				1	2	3	2	3
	4	3	2	4	3	3	5	4	4	3	1	2	2
	3	2	3	2	1	2	3	2	3	2	2	2	2
				2		2		2	2	2		2	
	2		2		2	2							
1	1	19	3	2	1	3	4	1	2	2	2		4
	2	2	2	2	3				2	2	2	1	4
	4	3	3	3	4	4	3	4	3	2	1	2	2
	2	2	3	3	2	3	2	3	3	2	2	2	2
				2		2		2	2	2		2	
	2		2		2	2							
2	1	36	2	3	1	1	11	1	1	3	1	1	4
	2	2	2	2	3				3	4	3	3	3
	5	3	4	3	3	3	2	3	3	1	4	3	2

	2	2	2	5	4	3	5	2	3	2	2	2	2
				2		2		2	2	2		2	
	2		2		2	2							
2	1	21	2	2	1	3	2	1	2	3	2		2
	2	2	2	2	3				1	2	2	2	4
	4	4	2	4	5	5	3	3	3	2	3	2	2
	1	1	2	2	1	3	1	3	3	1	2	2	2
	2	2	2	2	2	2	2		1		1		4
		6		2		2		2	2	2		2	
	2		2		2	2							
2	1	21	2	2	1	3	4	2	2	1	2		7
	2	2	2	2	3				3	2	1	2	3
	4	2	3	3	3	3	3	4	2	3	1	3	2
	1	3	2	2	3	3	5	3	2	2	2	2	2
				2		2		2	2	2		2	
	2		2		2	2							
2	2	23	2	2	1	3	4	1	2	1	2		7
	2	2	2	2					2	2	2	2	3
	5	4	3	2	4	3	3	5	3	2	4	2	2
	2	2	3	2	2	3	2	2	1	1	2	1	2
	2	2	2	2	2	2	1		1		1		1
		6		2		2		2	2	2		2	
	2		2		2	2							
1	1	21	2	2	1	3	4	2	2	1	2		4
	2	2	2	2	3	6	5		2	4	2	2	3
	3	2	3	4	2	2	3	4	3	1	4	3	3
	4	4	2	3	2	2	2	3	2	2	2	2	2
	2												
				2		2		1	1	2		2	
	2		2		2	2							
2	1	20	2	2	1	3	2	1	2	1	2		10
	2	2	2	2	3	6	2	1	2	2	4	3	3
	3	2	3	2	3	3	3	3	3	1	5	3	3
	2	2	3	4	2	3	2	2	1	1	1	2	2
	1	1	1	1	2	2	3	2	1	1	4	4	1
	1	6	6	2		2		2	2	2		2	
	2		2		2	2							
1	5	27	3	1	1	3	4	1	1	2	1	1	3
	2	2	2	2	3				1	1	1	1	4
	5	4	4	3	4	5	3	4	3	2	2	2	2
	1	2	1	2	1	1	1	2	3	2	2	2	2
	2	2	2	2	2	2				1	1	1	6
	6	6	6	2		2		2	2	2		2	
	2		2		2	2							
1	1	22	2	3	1	3	4	1	2	2	2		4
	2	1	2	1	1	7	2	2	2	3	4	2	3
	3	2	2	2	2	1	3	4	3	1	4	5	4
	5	2	2	2	3	3	1	2	5	1	1	1	2
	1	2	2	2	2	2	1		3		4		1
		5		2		2		2	2	2		2	
	2		2		1	2							
1	2	22	2	2	1	3	4	1	2	1	2		3
	2	2	2	1	2				2	1	1	2	3
	3	2	3	3	3	2	3	2	3	2	2	3	4

	3	3	2	3	2	3	2	2	3	1	2	1	2
	1	2	1	2	1	2	1		2		3		1
		4		1	4/5	2		2	2	2		2	
	2		2		2	2							
2	1	21	3	2	1	3	4	1	2	1	2		4
	2	2	2	2	3				3	4	3	1	3
	4	3	4	3	3	4	1	4	2	4	2	3	3
	3	3	2	3	4	4	4	2	2	2	2	2	2
	1	2	2	2	2	2	1	1	1	1	1	1	1
	1	6	6	2	0	2	0	2	2	2	0	2	0
	2	0	2	0	2	2							
2	1	37	2	2	1	3	4	1	2	3	1	2	5
	1	2	1	2	3				1	1	2	1	3
	4	4	4	2	4	4	3	4	3	2	3	2	2
	2	2	2	3	1	2	2	3	3	2	2	2	2
				2		2		2	2	2		2	
	2		2		2	2							
1	1	30	1	1	1	3	5	1	1	3	1	2	4
	2	2	2	2	3				2	3	3	3	3
	4	3	3	3	3	3	3	3	3	2	4	3	2
	2	3	2	3	2	3	1	3	5	1	2	2	2
	2	2	2	2	2	2	1		1		1		1
		6		2		2		2	2	2		2	
	2		2		2	2							
2	1	20	3	2	1	3	4	3		3	2		4
	2	2	2	1	2				2	4	3	3	2
	3	3	2	4	3	1	3	4	3	1	4	2	2
	5	3	5	2	2	2	2	2	1	1	2	1	2
	1	2	1	2	1	2	4		1		2		4
		4		2		2		2	2	2		2	
	1	2	2		1	2							
1	1	20	2	3	1	3	4	2	2	2	2		4
	2	2	2	1	2		5	1	2	2	1	2	3
	5	4	3	4	3	5	5	4	3	2	2	3	2
	2	3	3	3	2	2	1	3	2	1	1	2	2
	2	2	2	2	2	2	2		1		1		1
		4		2		2		2		2			
	2		2		2	2							
1	1	40	1	4	6	1	11	1	4	3	2		3
	2	2	2	2	3				1	2	3	2	3
	4	4	4	4	4	3	4	4	3	2	3	2	1
	2	2	2	2	2	2	2	3	3	2	2	2	2
				2	0	2	0	2	2	2	0	2	0
	2	0	2	0	2	2							
1	1	20	2	2	1	3	4	2	2	1	2		2
	2	2	1	1	2				2	2	2	3	2
	3	3	3	2	3	3	3	4	3	1	4	3	3
	2	3	2	2	3	2	3	3	3	1	2	2	2
	2	2	2	2	2	2							
				2		2		2	2	2		2	
	2		2		2	2							
1	1	39	1	2	3	1	11	1	4	3	2		1
	2	2	2	2	3				1	4	4	4	4
	4	2	3	4	3	3	3	4	4	2	3	4	4

	3	2	2	2	1	4	4	4	1	1	2	2	2
	2	2	1	2	2	2				1	1	1	6
	6	6	6	2		2		1	1	2		2	
	2		2		2	2							
1	3	22	1	1	1	3	6	2	1	3	2		2
	2	2	2	2	3				2	2	2	1	3
	5	4	4	4	3	4	4	4	4	2	2	2	2
	2	3	2	3	1	1	1	3	3	2	2	2	2
				2		2		2	2	2		2	
	2		2		2	2							
1	1	21	1	3	1	3	6	1	1	1	2		3
	2	1	2	2	3				3	3	2	2	3
	3	3	2	2	3	3	3	3	3	1	4	3	3
	4	2	2	3	5	4	2	4	1	1	2	2	2
	2	2	2	2	2	2	1				1		6
		5		2		2		2	2	2		2	
	2		2		2	2							
1	1	21	1	2	1	3	6	1	1	1	2		4
	2	2	2	2	3				3	3	1	1	3
	4	4	3	3	3	4	3	4	2	1	1	3	3
	3	4	3	2	1	1	4	4	2	2	2	2	2
	2	2	2	2	2	2							
				2		2		2	2	2		2	
	2		2		2	2							
1	1	21	1	2	1	3	6	1	1	1	2		4
	2	1	2	2	2	5			3	2	1	1	2
	4	3	3	3	3	4	2	4	2	2	2	3	3
	2	2	2	2	2	3	4	3	3	2	2	2	2
	2	2	2	2	2	2							
				2		2		2	2	2		2	
	2		2		2	2							
1	1	23	1	2	1	3	6	1	3	2	2		4
	2	2	1	1	2				2	3	3	3	3
	5	3	3	3	3	3	3	3	2	1	2	3	3
	3	3	3	3	5	3	4	3	1	1	2	1	2
	1	2	2	2	2	2	1		1		2		1
		5		1	1	2		2	2	2		2	
	1	1	2	0	2	2	43151	2	1	43151	1	1	
	43151	1	1										
1	3	24	2	3	1	3	6	2	3	3	2		3
	2	2	2	2	3				1	3	3	2	3
	5	3	4	4	4	3	2	4	3	1	3	2	1
	4	3	1	3	2	1	2	4	3	2	2	2	2
				2		2		2	2	2		2	
	2		2		2	2							
1	1	20	2	2	1	3	4	1	1	2	2		5
	2	2	2	2	3				2	1	1	1	5
	5	4	4	4	5	5	4	5	3	2	2	2	2
	1	1	1	1	1	2	1	3	3	2	2	2	2
	2	2	2										
				2		2		2	2	2		2	
	2		2		2	2							
2	2	31	2	2	1	3	6	2	2	2	2		1
	2	2	2	2	3				3	3	2	1	3
	4	3	4	4	3	4	3	3	2	2	3	2	2

	2	2	4	2	2	2	5	3	3	2	2	2	2
				2		2		2	2	2		2	
	2		2		2	2							
1	3	21	2	3	1	3	6	3		2	2		2
	2	2	2	1	2				1	3	2	2	4
	3	4	2	3	3	2	5	5	3	2	4	3	4
	4	2	2	1	2	2	2	3	2	1	1	1	1
	1	1	1	2	1	2	3	3	1	1	1	1	3
	3	4	4	2		2		1	2	2		2	
	2		2		1	2	41041	2	3	41041	2	3	
	41041	2	3										
1	1	23	2	2	1	3	6	2	1	1	2		2
	2	2	2	1	2				2	3	2	2	3
	3	2	3	4	3	3	3	4	3	1	3	3	3
	3	2	3	2	2	3	3	3	1	1	2	1	2
	1	2	1	2	2	2	1		1		2		4
		4		2		2		1	2	2		2	
	1	1	2		1	2							
2	1	23	4	2	1	3	5	3		1	2		2
	2	1	2	2	3				3	4	3	1	3
	3	2	2	4	3	4	4	5	5	1	4	2	2
	2	2	3	1	4	2	4	2	3	1	2	1	2
	2	2	2	2	2	2	1		1		1		1
	6	5	6	2		2		2	2	2		2	
	2		2		1	2							
2	1	22	2	2	1	3	6	1	2	1	2		3
	2	2	2	2	3				1	1	1	2	3
	5	4	3	3	4	3	4	4	4	2	4	2	2
	1	2	2	2	1	2	2	4	3	1	2	2	2
	2	2	2	2	2	2	1		1		1		6
	6	6	6	2		2		2	2	2	0	2	
	2		2		2	2							
1	3	22	3	3	1	3	6	3		1	2		6
	2	1	2	2	3				3	3	2	1	3
	3	3	2	3	3	3	2	3	2	2	3	3	3
	3	3	2	4	4	2	5	4	3	1	2	1	2
	2	2	2	2	2	2	1		3		3		4
		4		2		2		1	2	2		2	
	2		2		2	2							
1	1	34	1	1	1	3	6	1	2	2	1	1	3
	2	2	2	2	2				2	2	2	2	4
	4	3	4	4	3	4	3	3	3	1	3	3	3
	2	2	2	2	2	3	2	2	3	2	2	2	2
				2		2		2	2	2		2	
	2		2		2	2							
2	3	25	1	1	1	3	6	1	2	2	2		3
	2	2	2	2					3	4	3	1	2
	2	2	3	3	3	3	2	4	2	1	2	3	2
	3	1	2	2	4	3	5	4	5	1	1	1	2
	1	2	2	2	2	2	1		1		4		1
		5		2		2		1	2	2		2	
	2		2		2	2							
1	3	24	2	2	1	3	6	1	1	1	2		4
	2	2	2	2	3				3	3	3	3	3
	4	3	3	3	3	5	2	3	3	2	2	3	2

	2	2	2	2	2	2	5	2	3	2	2	2	2
				2		2		2	2	2		2	
	2		2		2	2							
1	1	31	2	1	1	3	6	3		2	1	1	3
	2	2	2	2	3				1	2	2	2	4
	4	3	4	4	3	4	3	4	3	1	1	2	2
	2	2	2	1	1	2	1	2	3	1	2	2	2
	2	2	2	2	2	2	1		1		1		6
		5		2		2		2	2	2		2	
	2		2		2	2							
2	1	22	2	2	1	3	6	1	1	2	2		4
	2	2	2	2	3				1	1	1	1	4
	5	4	4	4	4	5	3	5	3	2	3	3	3
	2	2	1	2	2	2	2	2	3	2	2	2	2
				2		2		2	2	2		2	
	2		2		2	2							
2	1	23	3	3	1	3	6	2	2	4	2		5
	2	2	2	2	3				1	2	3	2	3
	3	4	3	4	3	3	4	5	3	2	4	3	3
	4	4	1	4	2	2	2	4	3	1	2	1	2
	1	2	1	2	1	2	1		5		3		4
		4		2				1	2	2			
	2				2								
2	1	21	2	2	1	3	6	1	1	1	2		5
	2	2	2	2	3				2	2	2	2	4
	3	3	3	3	4	4	3	5	3	1	2	3	2
	2	3	2	2	2	2	3	3	2	2	2	2	2
				2				2	2	2		2	
	2		2		2	2							
1	1	22	2	3	1	3	6	1	2	2	2		3
	2	2	2	2	3				2	3	2	2	3
	4	3	3	4	3	2	3	4	2	2	2	3	2
	3	3	3	3	2	2	3	3	3	1	2		
					2	2			1		1		
		6		2		2		2	2				
	2				2	2							
1	1	24	2	2	1	3	7	2	1	1	2		6
	2	2	2	1	2				2	2	1	3	2
	4	1	2	3	2	5	2	3	3	2	4	4	3
	4	2	2	2	2	2	3	2	1	1	1	1	1
	2	2	1	2	2	2	1		1	1	3	2	1
	1	4	4	2		2		2	2	2		2	
	2		2		2	2							
1	1	21	2	2	1	3	6	3		1	2		4
	2	1	2	1	1	4	5	2	4	3	2	2	2
	4	4	2	2	4	3	2	3	2	2	2	4	4
	4	3	3	2	5	2	4	5	3	1	2	1	2
	2	2	1	2	2	2	1		1		1		1
		4		2				1	1	2		2	
	2		2		2	2							
2	3	20	3	2	1	3	2	2	3	1	2		4
	2	2	2	1	2				3	2	2	2	2
	3	2	2	3	1	3	1	5	2	2	3	4	4

	5	5	5	5	3	4	2	4	5	1	2	1	2
	1	2	2	2	2	2	2		2		4		3
		4		2		2		1	1	2		2	
	1	1	2		2	2							
1	1	22	2	3	1	3	6	1	2	1	2		3
	2	2	2	1	2				2	2	1	1	3
	4	3	3	4	3	3	3	3	3	2	2	2	2
	2	1	2	2	1	4	2	2	3	1	2	1	2
	1	2	1	2	2	2	1		2		1		1
		4		2		2		2		2			
	1	3	2		1	2							
1	1	26	2	1	1	3	4	2	2	1	2		2
	2	2	2	1	2	6	2	1	2	2	1	1	2
	3	3	2	3	2	1	1	3	2	1	3	3	4
	4	4	4	4	4	3	3	3	2	1	2	1	2
	1	2	1	2	2	2	2		2		1		1
		5		2		2		2	2	2		2	
	1	3	2		1	2							
1	1	39	2	1	1	3	6	1	1	3	1	2	4
	2	2	1	1	1	8	7	1	2	3	1	4	3
	3	2	2	3	1	3	3	3	4	1	4	5	3
	2	2	2	2	2	3	2	4	1	1	1	1	1
	1	1	1	1	1	1	4	4	1	1	3	1	1
	1	6	6	2		2	0	2	2	1	1	2	0
	2		2		2	2							
1	1	24	3	3	1	3	7	2	1	1	2		5
	2	2	2	1	2				2	2	2	1	4
	3	3	3	4	2	3	1	2	2	2	5	5	5
	3	2	1	4	3	3	5	4	5	1	1	1	1
	1	1	2	2	2	2	2	1	1	1	2	2	1
	1	4	4	2				2	2	2		2	
	2		2		2	2							
2	1	22	1	3	1	3	7	1	2	1	2		2
	2	2	2	2	3				1	2	2	2	4
	4	3	3	3	4	4	3	4	3	2	3	2	2
	2	2	3	2	2	3	3	3	3	1	2	2	2
	2	2	2	2	2	2	1		1		1		1
		5		2		2		1	2	2		2	
	2		2		2	2							
1	3	22	1	2	1	3	7	3		2	2		4
	2	1	2	1	1	4	3	1	3	4	2	2	3
	2	2	2	3	2	2	2	3	3	1	4	4	5
	5	2	3	1	2	3	2	5	1	1	1	1	1
	1	1	1	1	2	2	4	4	3	2	6	3	1
	1	5	5	2		2		1	2	2		2	
	2		2		1	2							
1	1	22	2	2	1	3	6	2	2	1	2		3
	2	2	2	1	2				3	3	3	1	3
	3	3	3	3	3	4	3	4	3	1	3	3	3
	3	3	3	2	2	2	2	3	3	1	1	1	2
	2	2	2	2	2	2	1	1	2		1		3
		4		2		2		1	2	2		2	
	2		2		2	2							
1	1	22	2	3	1	3	7	1	2	2	2		4
	2	2	2	2	2				1	2	2	1	4
	5	3	4	4	5	5	3	5	5	2	2	1	1

	1	1	1	1	1	1	1	1	1	1	2	2	2
	2	2	2	2	2	2			1		1		1
		6	6	2		2		2	2	2		2	
	2		2			2							
1	1	23	2	3	1	3	7	1	2	1	2		5
	2	2	2	1	2				2	3	2	2	3
	4	4	3	3	3	3	2	4	3	2	3	3	2
	2	3	3	3	3	3	5	2	3	1	2	2	2
	1	2	1	2	2	2	1		1		2		1
		4		2		2		2	2	2		2	
	2		2		2	2							
2	1	22	2	3	1	3	6	1	2	1	2		7
	2	2	2	2	3		2	1	2	5	2	1	4
	5	4	4	4	5	5	3	4	3	2	2	2	2
	1	2	2	2	2	2	2	2	3	2	2	1	2
	2	2	1	2	2	2	1		1		1		6
	6	6	6	2		2		2	2	2		2	
	2		2		2	2							
2	1	22	3	2	1	3	7	1	3	3	2		3
	2	2	2	2	3				1	2	3	2	4
	5	2	3	4	3	5	3	4	4	2	2	2	2
	2	2	1	3	3	1	2	3	3	2	2	2	2
	2	2	2	2	2	2					3		
				2		2		2	2	2		2	
	2		2		2	2							
	4												
1	2	21	1	3	1	3	6	3		2	2		3
	2	2	2	1	2				4	4	2	2	3
	1	2	1	4	3	2	3	4	3	2	5	5	5
	3	3	3	4	3	3	1	5	5	1	2	1	2
	1	2	2	2	2	2	1		3		1		4
		5		2				1	1	2		2	
	2		2		2	2							
1	2	22	2	3	1	3	7	2	2	2	2		5
	2	2	2	2	3				2	4	2	1	3
	3	5	3	3	3	3	3	3	2	2	5	3	3
	4	3	4	3	2	3	5	3	5	1	1	1	1
	1	2	2	2	2	2	2		2		2		4
		5		2		2		1		2			
	1	3			1								
1	1	22	2	1	1	3	7	2	2	2	2		7
	1	2	2	1	2	6	2	1	2	4	3	3	2
	4	2	2	4	2	3	2	4	2	2	4	5	4
	3	2	2	2	2	3	3	3	3	1	2	1	2
	1	2	1	2	2	2	3		2		1		1
		4		2		2		1	1	2		2	
	2		2		1	2							
1	1	19	2	2	1	3	4	1	3	2	2		5
	2	2	2	1	2				2	4	2	2	4
	4	3	3	3	2	3	3	4	3	1	2	2	2
	2	3	4	3	2	2	2	3	2	1	1	1	2
	2	2	1	2	2	2	1		2		2		1
		4		2		2		1	2	2		2	
	1		2		1	2							
1	1	22	2	2	1	3	7	2	3	1	2		4
	2	2	2	2	2				4	4	4	3	3
	2	2	2	1	2	3	2	3	1	4	3	4	4

	4	2	2	3	5	3	5	3	1	1	1	2	2
	2	2	2	2	2	2	1	2	3	3	2	1	1
	1	6	6	2		2		2	2	2		2	
	2		2		2	2							
2	2	22	1	2	1	3	5	3		2	2		5
	2	2	1	1	2				2	3	3	2	3
	3	4	4	2	3	2	3	4	3	2	2	3	3
	4	4	2	3	2	3	4	4	1	1	2	1	2
	1	2	1	2	1	2	4		2		6		6
		5		1	2	2	0	2	2	1	2	2	0
	2	0	2	0	2	2	42750	1	3	42750	5	3	
	42658	3	2										
2	1	30	2	1	1	3	4	2	1	2	1	1	4
	2	2	2	2	3				2	5	1	2	3
	4	3	4	2	3	5	3	3	2	2	3	2	2
	2	2	1	3	3	2	2	2	3	2	2	2	2
				2		2		2	2	2		2	
	2		2		2	2							
2	1	20	2	2	1	3	2	1	1	1	2		5
	2	2	2	1	2				2	3	1	1	2
	3	2	2	4	4	4	3	3	3	2	4	4	4
	4	3	3	3	3	3	2	3	1	1	1	1	2
	1	2	2	2	2	2	3	3	3	2	3	3	1
	1	5	5	2		2		1	1	2		2	
	1		1		2	2							
1	1	19	3	2	1	3	4	1	1	3	2		3
	2	2	2	2	3				1	2	1	1	3
	4	4	4	3	4	4	3	3	4	2	3		2
	2	2	3	2	1	3	2	2	3	2	2	2	2
				2		2		2	2	2		2	
	2		2		2	2							
2	1	20	2	2	1	3	2	3		1	2		4
	2	2	2	1	2				3	3	2	1	2
	2	1	3	3	1	2	2	2	3	3	5	5	5
	5	5	5	5	3	3	5	4	2	1	1	1	1
	1	2	1	2	1	2	2	1	3	1	3		4
		4	6	1	1	2		2	2	2		2	
	1	1	2		1	2	44070						
2	1	27	1	3	1	3	4	2	2	4	2		5
	1	2	2	2	3		2	1	2	3	4	2	2
	3	1	3	3	5	5	3	5	4	4	4	4	3
	3	2	3	3	3	3	1	4	5	2	2	2	2
	1	2	1	2	2	2							
				2		2		1	2	1	1	2	
	2		2		2	2							
1	1	32	2	1	1	3	8	3		1	1	1	3
	2	2	1	1	1	3	2	1	3	2	2	2	2
	3	3	2	3	2	1	2	3	2	4	4	4	4
	4	3	2	3	2	2	3	2	3	1	2	1	2
	1	2	1	2	1	2	5		4				4
		5		1	2	2	0	1	2	1	1	2	0
	1	1	2	0	1	2							
1	1	32	2	1	1	3	8	3		1	1	1	3
	2	2	1	1	1	3	2	1	3	2	2	2	2
	3	3	2	3	2	1	2	3	2	4	4	4	4

	4	3	2	3	2	2	3	2	3	1	2	1	2
	1	2	1	2	1	2	5		4				4
		5		1	2	2	0	1	2	1	1	2	0
	1	1	2	0	1	2							
2	2	22	3	2	1	3	7	3		1	2		4
	2	2	2	1	2				2	3	2	1	3
	4	4	3	4	3	3	3	4	2	2	2	2	2
	3	3	4	2	2	2	3	3	3	1	2	1	2
	1	2	2	2	2	2	1		1		1		1
		4		2		2		2		2			
	2				2								
2	1	23	4	3	1	3	7	1		2	2		3
	2	2	1	2	3				1	2	1	3	4
	5	4	4	3	3	4	3	4	3	2	2	2	2
	2	1	3	1	1	2	1	2	3	2	2	2	2
				2		2		2		2		2	
			2		2	2							
1	1	21	2	3	1	3	7	1	2	2	2		4
	2	2	2	2					1	2	1	2	4
	5	5	4	5	4	5	5	5	4	2	2	2	1
	1	1	2	1	1	2	1	2	3	2	2	2	2
				2		2		2	2	2		2	
	2		2		2	2							
1	1	19	3	2	1	3	2	1	1	2	2		5
	2	2	2	2	3				2	2	2	2	3
	4	3	3	2	3	3	2		3	1	4	3	3
	4	4	3	2	2	2	4	4	2	2	2	2	2
				2		2		2	2	2		2	
	2		2		2	2							
1	1	32	2	1	1	3	6	1	1	2	1	1	3
	2	2	2	2	3				1	1	1	2	4
	4	4	3	4	4	4	3	4	4	2	2	2	2
	2	2	1	2	2	2	2	4	3	2	2	2	2
				2		2		2	2	2		2	
	2		2		2	2							
2	2	21	2	2	6	3	5	1	2	2	2		4
	2	2	2	2	3				2	2	1	1	4
	3	4	3	3	3	5	3	3	4	2	3	3	3
	4	3	3	2	3	2	3	3	3	2	2	2	2
				2		2		2	2	2		2	
	2		2		2	2							
2	1	19	2	2	1	3	2	1	3	2	2		3
	2	2	2	1	2		5	1	1	2	1	2	4
	4	3	4	4	3	3	3	4	3	2	2	2	3
	2	2	2	1	2	2	2	3	3	1	2	2	2
	1	2	2	2	2	2	1	1	1		1	1	1
	1	6	6	2		2		2	2	2		2	
	2		2		2	2							
2	1	23	2	1	1	3	7	1	2	1	2		4
	2	2	2	2	3				3	4	3	2	2
	4	1	3	4	3	4	2	3	2	1	4	4	4

	4	1	2	1	4	3	4	5	2	2	2	2	2
				2		2		2	2	2		2	
	2		2		2	2							
1	3	18	1	3	1	3	2	1	1	3	2		3
	2	2	2	1	2				2	2	2	3	3
	2	3	3	4	3	3	3	4	5	2	3	4	4
	4	2	2	2	2	2	2	2	1	1	2	1	2
	1	2	1	2	1	2	2		3		6		4
		3		1	1	2		1	1	2		2	
	1	1	2		1	2							
1	1	20	2	3	1	3	2	1	2	1	2		4
	2	2	2	1	1	3	2	1	3	4	2	2	3
	3	2	1	2	3	2	3	3	3	1	5	2	4
	4	4	2	5	2	3	4	2	3	1	1	1	1
	1	2	1	2	1	2	5		5		4		5
		3		1	2	2		1	2	1	2	2	
	2		2		1	2	44423	1	2	44410	2	3	
	44410	2	3										
2	1	38	1	1	1	1	11	1	10	4	1	2	4
	2	2	2	2	3				1	2	1	2	4
	5	4	4	4	4		5	4	3	2	2	2	2
	1	2	2	1	3	2	1	2	3	2	2	2	2
				2		2		2	2	2		2	
	2		2		2	2							
2	1	38	1	1	12	1	11	2	1	4	1	1	3
	2	2	2	2	3				2	2	3	2	3
	5	3	4	4	4	4	3	4	3	2	2	2	2
	2	3	3	3	2	3	2	3	1	2	2	2	2
				2		2		2	2	2		2	
	2		2		2	2							
2	1	24	3	2	6	3	6	3		4	2		5
	2	2	2	1	1	9	8	1	2	4	4	3	2
	2	2	1	3	3	2	1	3	3	1	5	4	5
	5	3	4	1	1	2	2	4	1	1	2	1	2
	1	2	1	2	1	2	4		3		2		4
		5		1	5	2		2	2	2			
	1	5	2		1	2	44148	1	3	44148	1	3	
	43474	1	3										
1	1	22	1	2	6	3	8	2	1	2	2		2
	2	1	1	1	2				2	2	2	1	3
	3	3	3	4	3	2	3	4	3	2	3	3	3
	3	2	2	2	2	2	2	3	3	1	2	1	2
	1	2	1	2	2	2	1		2		3		1
		3		2		2		2	2	2		2	
	2		2		2	2							
2	2	37	3	3	6	3	2	1	1	2	2		3
	2	2	2	1	1	6	9	2	2	2	1	4	3
	3	3	3	4	3	4	3	5	5	2	2	3	2
	3	3	1	2	2	4	4	3	1	1	2	1	2
	1	2	1	2	1	2	5		5		6		1
		5		2		2		1	1	2		2	
	1	10	2		1	2							
2	2	25	3	2	6	3	5	3		1	2		2
	2	2	2	1	2				2	2	1	2	4
	4	3	3	3	3	4	3	3	3	1	2	2	3

	2	2	2	3	3	3	2	3	1	2	2	1	2
	1	2	2	2	1	2	2		1		1		1
		6		2		2		1		2			
	2				2	2							
1	1	20	2	2	6	3	4	1	10	1	2		3
	2	1	2	2	3	4			2	2	2	2	3
	3	3	2	3	1	2	3	4	4	3	4	4	4
	4	4	3	3	2	4	3	5	5	1	2	1	2
	1	2	1	2	1	2	1		1		5		4
		5		2		2		2	2	2		2	
	2		2		2	2							
1	1	22	2	3	1	3	6	1	2	2	2		4
	2	2	2	2	3				1	2	1	2	4
	5	3	4	4	4	5	5	4	4	2	3	3	2
	2	2	2	2	2	2	2	3	3	2	2	2	2
	2	2	2	2	2	2							
				2		2		2	2	2		2	
	2		2		2	2							
1	3	24	1	3	6	3	6	3		2	2		3
	2	1	2	1	1	3	6	1	2	2	3	3	3
	3	2	3	2	2	4	2	4	3	2	4	4	3
	3	3	3	3	3	4	2	3	5	1	1	1	1
	1	1	1	1	2	2	4	2	4	2	4	2	4
	5	5	5	2		2		2	2	2		2	
	2		2		1	2							
1	1	38	1	4	6	3	6	1	2	3	2		1
	2	2	2	1	1	3	2	1	3	3	3	3	3
	3	3	3	3	3	3	3	4	3	2	4	3	3
	3	2	3	2	3	3	1	3	2	1	2	1	2
	2	2	1	2	1	2	1		1		3		1
		4		2		2		1	1	2		2	
	2		2		2	2							
2	2	29	2	3	6	3	6	2	3	2	2		4
	2	2	2	2	2				2	3	1	1	3
	3	4	2	3	3	3	3	4	3	1	2	3	2
	3	2	2	3	1	3	2	4	3	1	2	2	2
	2	2	2	2	2	2	1		1		1		6
		6		2				2	2	2		2	
	2		2		2	2							
2	1	21	1	2	6	3	6	1	1	3	2		4
	2	2	2	2	3				2	2	2	2	2
	4	3	4	4	3	3	3	5	3	3	4	4	3
	5	4	5	3	2	1	3	4	1	1	2	2	2
	2	2	2	2	2	2	1		1		3		1
		5		2		2		1	2	2		2	
	2		2		1	2							
2	1	23	2	2	6	3	4	2	2	2	2		1
	2	2	2	2	3				2	2	1	2	5
	4	4	3	3	3	3	3	4	3	2	4	3	3
	2	4	2	3	3	3	4	4	3	2	2	2	2
	2	2	2	2	2	2					1	1	6
	6	6	6	2		2		1	1	2		2	
	2		2		2	2							
2	1	21	4	2	6	3	5	1	1	3	2		4
	2	2	2	2	3				2	3	2	2	4
	4	3	4	3	4	3	2	3	4	1	2	2	2

	2	2	3	2	2	4	2	2	3	2	2	2	2
	2	2	2	2	2	2							
				2		2		2	2	2		2	
	2		2		2	2							
1	1	24	2	1	1	3	7	1	2	2	2		4
	2	2	2	2	3				3	2	3	1	3
	5	4	4	3	3	3	2	4	3	2	4	3	3
	3	3	2	2	2	2	2	3	3	2	2	2	2
				2		2		2	2	2		2	
	2		2		2	2							
2	1	26	2	2	6	3	6	3		4	2		3
	2	2	2	2	3				2	4	3	3	2
	4	4	4	4	3	4	4	5	4	1	4	2	2
	2	2	2	2	2	2	1	4	3	1	2	1	2
	1	2	1	2	2	2	1		5		2		4
		5		2				2		2			
	2				1	2							
2	1	23	1	2	6	3	7	3		3	2		4
	2	2	2	2	3				2	2	2	2	4
	5	4	4	3	3	5	3	3	3	2	1	2	3
	2	2	2	3	2	3	2	3	3	2	2	2	2
				2		2		2	2	2		2	
	2		2		2	2							
2	3	20	3	3	6	3	4	3		2	2		2
	2	1	1	1	1	8	2	2	3	4	2	2	3
	3	2	2	2	3	2	4	5	2	2	4	3	2
	4	4	2	2	3	2	2	4	5	1	2	1	2
	1	2	1	2	1	2	5		3		4		5
		5		1	2	2		1	2	2		2	
	1	2	2		1	2	43377	1	3	43442	1	3	
	43377	1	3										
2	2	48	2	1	6	3	7	2	1	3	1	6	3
	2	2	2	1	2		5	1	2	4	5	4	4
	5	3	4	3	3	2	3	4	2	3	4	4	4
	3	2	2	4	4	4	4	2	3	2	2	1	2
	2	2	2	2	2	2	1		1		2		1
		4		2		2		2	2	2		2	
	2		2		2	2							
1	1	21	2	2	6	3	4	1		1	2		3
	2	2	2	2	3				3	3	3	2	2
	3	3	2	3	3	2	1	3	1	3	3	4	3
	3	3	3	2	3	3	4	4	2	2	2	2	2
				2		2		2	2	2		2	
	2		2		2	2							
2	1	25	3	2	6	3	5	3		2	2		5
	2	2	1	1	1	1	6	1	2	3	1	3	2
	3	3	3	3	3	2	2	4	2	1	4	2	4
	4	2	5	5	4	3	2	3	3	1	2	1	2
	1	2	1	2	1	2	4		4		6		1
		5		1	2	2		2		1	1		
	1	1			1		44294	1	3	44294	1	3	
	43892	1	3										
2	3	21	3	2	6	3	6	3		1	2		4
	2	2	2	1	1	10	6	2	3	3	4	3	3
	2	2	2	2	2	2	2	2	2	3	5	5	5

	5	2	5	3	3	3	5	5	1	1	1	1	2
	1	2	1	2	1	2	5	1	5	2	6	3	5
	5	5	5	1	4	2		1	2	2		2	
	1	4	2		1	2	43874	2	3	43874	2	2	
	43321	3	2										
2	3	19	2	2	6	3	4	3		1	2		1
	2	1	1	1	2				2	3	3	2	3
	4	3	4	4	3	3	2	4	4	1	2	2	2
	2	4	2	4	2	2	1	2	2	1	2	1	2
	1	2	2	2	2	2	1		2		3		4
		3		1	1	2		2	2	2		2	
	2		2		1	2	41713	1	3	41713	1	3	
	41713	1	3										
1	3	21	2	3	6	3	2	2	2	1	2		10
	2	2	2	1	2				3	4	4	3	3
	2	2	2	2	2	3	2	3	2	1	4	4	3
	3	3	3	3	5	3	4	2	2	1	2	1	2
	2	2	2	2	2	2	1		1		1		1
		5		2		2		1	2	2		2	
	1	3	2		2	2							
2	1	39	1	2	6	3	4	1	2	2	1	4	5
	1	2	2	1	2	6	2	1	1	1	1	3	4
	5	3	4	2	4	5	3	4	2	1	3	2	2
	2	4	1	2	2	3	2	2	3	1	2	1	2
	1	2	1	2	1	2	1		5		4		1
		4		1	2			2	2	1	1	2	
	1	1	2		1								
2	1	33	2	1	6	3	4	1	3	1	1	2	4
	1	2	1	2	3	6	5	1	1	2	3	2	4
	4	3	3	2	4	4	2	3	3	1	2	3	2
	1	2	1	3	3	3	3	2	2	2	2	2	2
	2	2	2	2	2	2							
				2		2		1	1	2		2	
	2		2		2	2							
1	1	26	2	2	6	3	4	2	1	2	2		2
	2	2	2	2	3				2	3	2	3	3
	4	3	3	4	3	2	3	3	3	2	3	3	3
	3	3	3	4	2	3	2	2	1	1	1	2	2
	2	2	2	2	2	2			1		2		
		4		2		2		2	2	2		2	
					2	2							
2	1	39	2	2	6	3	8	1	1	4	1	2	4
	2	2	2	2	3				2	3	2	3	3
	4	3	4	3	3	4	3	4	3	1	3	2	2
	2	2	2	3	2	3	2	3	3	2	2	2	2
				2		2		2	2	2		2	
	2		2		2	2							
2	2	23	1	2	6	3	8	2	2	2	2		1
	2	1	2	1	2				2	3	3	3	3
	3	3	3	3	3	3	3	4	3	3	2	4	4
	4	3	3	3	3	2	4	4	3	1	1	1	1
	1	2	2	2	2	2	4		3	1	3	1	4
	3	4	6	2		2		2	2	2		2	
	2		2		2	2							
1	1	30	2	4	6	3	4	1	2	1	2		6
	2	2	2	1	2				2	3	3	2	3
	4	2	2	2	3	3	2	3	2	1	4	3	3

	3	2	3	2	2	4	2	3	3	1	2	1	2
	1	2	2	2	2	2	1		2		3		4
		4		2		2		2	2	2		2	
	2		2		1	2							
2	3	24	4	2	6	3	8	1	2	2	2		3
	2	2	2	2	3				2	3	1	3	4
	3	4	4	4	3	4	2	4	3	3	1	2	2
	2	2	3	2	2	3	4	3	2	2	2	2	2
				2		2		2	2	2		2	
	2		2		2	2							
2	1	23	2	3	6	3	8	1	1	2	2		5
	2	2	2	2	3				2	4	1	1	3
	5	4	2	4	3	3	1	5	4	2	3	4	
	4	4	2	1	2	4	4	1	3	2	2	2	2
	2	2	2	2	2	2							
				2		2		2	2	2		2	
	2		2		2	2							
1	1	21	2	3	6	3	8	2	1	4	2		5
	2	2	2	1	2				1	2	1	1	4
	5	3	4	4	4	5	4	5	4	2	2	2	2
	2	2	1	1	2	2	1	2	3	1	2	1	2
	1	2	1	2	2	2	3		1		3		1
		5		2		2		2	2	2			
	2				2	2							
1	1	22	2	2	6	3	4	2	1	2	2		2
	2	2	2	1	2				2	4	3	3	
	3	3	2	3	3	3	2	4	2	3	5	3	2
	4	4	2	2	3	3	3	4	1	1	1	1	1
	1	1	1	1	1	1	4	1	2	2	2	3	4
	4	4	4	1	2	2		1	2	1	1	2	
	1	1	2		1	2	42436	1	2	42937	3	3	
	42436	1	2										
2	1	45	2	1	6	3	8	2	1	3	1	1	3
	2	2	2	2	3				2	2	3	2	3
	4	4	4	4	4	4	4	4	4	2	2	2	2
	2	2	3	2	2	2	2	3	3	2	2	2	2
				2		2		2	2	2		2	
	2		2		2	2							
2	1	23	2	2	6	3	8	2	3	3	2		3
	2	2	2	2					3	4	3	2	3
	4	3	3	4	4	3	3	4	4	2	2	3	4
	4	3	2	2	2	3	2	4	3	1	2	1	2
	1	2	2	2	2	2	2		1		1		1
		6		2				2		2		2	
	2		2		2	2							
1	1	29	2	5	6	3	4	1	1	2	1	1	6
	2	2	2	2	3				2	2	1	1	3
	4	4	4	3	3	5	3	5	4	2	2	3	1
	3	1	1	1	4	2	2	2	3	2	2	2	2
				2		2		2	2	2		2	
	2		2		2	2							
2	1	20	1	2	6	3	4	1	1	3	2		2
	2	2	2	2	2				3	2	1	1	3
	3	3	3	3	3	3	3	4	3	3	3	3	2

	3	3	2	3	2	3	3	2	3	1	2	2	2
	2	2	2	2	2	2					1	1	6
	6	6	6	2		2		2	2	2		2	
	2		2		2	2							
2	1	21	2	2	6	3	5	1	2	2	2		4
	2	2	2	2	3				3	2	2	2	3
	5	3	3	3	4	3	2	4	3	4	4	3	3
	4	4	4	4	4	2	4	4	2	2	2	2	2
				2		2		2	2	2		2	
	2		2		2	2							
2	1	23	2	3	6	3	5	1	2	2	2		5
	1	1	2	2	3				3	4	2	3	2
	2	3	3	3	3	2	2	3	2	3	4	3	3
	3	3	4	3	3	4	4	3	3	2	2	2	2
	2	2	2	2	2	2							
				2	0	2	0	2	2	2	0	2	0
	2	0	2	0	2	2							
1	1	27	2	2	6	3	2	2	1	1	1	1	5
	2	2	2	2	3	6	5	1	2	1	3	1	4
	5	4	4	3	4	4	2	3	3	1	3	2	1
	1	2	3	3	2	4	4	1	3	2	2	2	2
				2		2		2	2	2		2	
	2		2		2	2							
2	1	38	2	2	6	3	8	2	2	3	1	2	10
	2	2	1	1	2				2	2	3	3	3
	4	4	4	3	3	4	3	4	3	1	3	2	4
	2	2	2	3	3	5	2	5	2	1	2	2	2
	1	2	1	2	2	2	2		1		1		1
		4		2	0	2	3	2	2	2	0	2	0
	2	0	2	0	2	2							
1	1	20	2	3	3	3	4	1	1	1	2		7
	2	2	2	2	3		3	1	2	2	4	2	3
	4	3	3	3	4	3	3	3	2	1	3	2	3
	2	2	1	3	3	3	2	3	3	1	2	2	2
	2	2	2	2	2	2	1		1		1		6
		4		2				2	2	2		2	
	2		2		2	2							
1	1	25	1	3	3	3	10	1	3	2	2		3
	2	2	2	2	3				3	2	1	1	2
	2	2	2	3	3	3	2	2	2	1	4	4	3
	4	3	2	4	3	4	2	5	5	1	1	1	1
	2	2	2	2	2	2	1		1		1		1
		5		2		2		1	2	2			
	2		2		2	2							
2	1	26	2	2	3	3	8	1	1	1	2		5
	2	2	2	1	2				2	2		2	4
	4	3	4	3	4	5	3	3	3	2	4	2	2
	1	1	3	3	3	3	4	2	3	1	2	1	2
	2	2	2	2	2	2	1		4		1		1
		4		2				2		1	1		
	2				2								
2	1	25	2	2	3	3	10	3	1	2	1	1	6
	2	2	2	2	3			1	1	2	4	3	3
	4	4	4	3	5	5	3	3	2	2	2	2	1

	1	3	3	2	2	3	3	2	1	2	2	2	2
		2	2	2									
				2		2		2	2	2		2	
	2		2		2	2							
2	3	22	4	2	3	3	10	2	2	1	2		2
	2	2	1	2	3				1	4	1	2	4
	2	1	3	4	1	1	3	5	4	1	2	5	5
	5	3	3	3	1	2	3	3	5	2	2	2	2
				2		2		2	2	2		2	
	2		2		2	2							
2	1	24	2	2	3	3	2	1	1	1	2		3
	2	2	2	2	3				2	1	1	2	3
	4	4	4	3	4	4	2	3	3	1	1	2	2
	2	2	2	2	3	3	4	2	4	2	2	2	2
				2		2		2		2		2	
	2		2		2	2							
1	1	19	2	3	3	3	1	1	2	1	2		2
	2	2	2	2	3				1	4	1	2	5
	5	4	4	4	3	3	3	4	4	1	2	2	2
	2	1	2	2	2	3	3	2	4	2	2	2	2
				2		2		2	2	2		2	
	2		2		2	2							
1	3	20	2	3	6	3	4	1	1	1	2		1
	2	2	2	2	3				2	3	4	1	4
	4	3	3	3	3	3	3	4	3	3	2	3	2
	3	2	1	2	4	2	4	5	3	1	2	2	2
	2	2	2	2	2	2	1		1		1		1
		5		2		2		2	2	2		2	
	2		2		2	2							
1	1	21	3	3	3	3	6	2	9	2	2		6
	2	2	2	2	3				1	4	2	2	1
	2	4	3	3	2	3	3	4	2	1	1	5	3
	3	2	3	1	3	3	3	3	2	1	2	2	2
				2		2		2	2	2		2	
	2		2		2	2							
1	1	23	2	3	3	3	9	1	2	1	2		6
	2	2	2	2	3				3	2	3	2	4
	4	3	3	3	3	5	3	3	4	1	2	2	2
	2	1	1	1	2	3	3	2	3	2	2	2	2
	2	2	2	2	2	2							
				2		2		2	2	2		2	
	2		2		2	2							
1	1	26	2	1	3	3	10	2	2	2	1	1	3
	2	2	2	2	3				1	2	1	2	4
	5	4	4	3	4	4	3	4	4	2	3	2	2
	1	1	1	1	2	2	1	2	2	1	2	2	2
	2		2		2		1		1		1		1
		4		2		2		2	2	2		2	
	2		2		2	2							
2	3	23	1	2	3	3	2	1	1	1	2		9
	2	2	2	2	3				1	1	1	1	5
	4	4	4	4	5	5	3	4	4	2	1	1	2

	1	1	2	2	2	2	2	2	3	2	2	2	2
				2		2		1	2	2		2	
	2		2		2	2							
2	1	24	2	2	3	3	8	1	2	2	2		8
	2	2	1	2					2	2	3	4	5
	5	4	4	3	4	5	2	3	2	1	2	2	2
	1	1	1	2	2	2	1	2	3	1	2	2	2
	2	2	2	2	2	2	1		1		1		6
	6	1		2		2		2	2	2		2	
	2		2		1	2							
1	1	20	2	2	3	3	6	1	2	1	2		4
	2	2	2	2	3		2	1	2	2	3	2	4
	5	3	4	3	2	4	3	3	3	1	4	3	3
	3	2	2	3	2	4	4	2	4	2	2	2	2
				2		2		2	2	2		2	
	2		2		2	2							
1	1	22	3	1	3	3	6	1	2	2	2		2
	2	2	2	2					2	2	1	4	4
	5	4	4	3	3	4	3	5	3	1	2	2	2
	3	1	1	1	2	3	2	4	3	2	2	2	2
				2		2		2	2	2		2	
	2		2		2	2							
2	1	23	2	3	3	3	10	2	3	2	2		7
	2	2	2	1	2				2	4	1	1	2
	3	3	2	4	3	3	3	5	2	1	4	2	2
	4	3	2	5	2	3	4	2	1	1	2	1	2
	1	2	2	2	2	2	3		1		2		1
		3		2		2		2	2	2		2	
	2		2		2	2							
2	1	21	2	2	3	3	4	2		1	2		4
	2	2	2	2	3				1	2		1	3
	4	4	4	4	3	4	3	4	1	1	2	2	
	1	2	3	2	1	2	1	2	4	2	2	2	2
				2		2		2	2	2		2	
	2		2		2	2							
2	1	26	2	2	3	3	10	3		1	2		1
	2	2	2	2	3				2	2	1	1	4
	4	4	4	4	4	5	3	3	3	1	2	2	2
	2	3	2	3	2	3	3	2	4	2	2	2	2
				2		2		2	2	2		2	
	2		2		2	2							
1	1	24	2	2	3	3	10	2	2	1	2		4
	2	1	1	1	2				2	3	3	4	3
	3	3	2	1	3	3	3	2	3	2	4	2	2
	4	2	3	3	5	5	3	2	3	1	1	1	2
	1	2	1	2	1	2	2		3		3		4
		4		1	1	2		1	2	2			
	2				2	2	43465	3	2	43465	3	2	
	43465	3	2										
1	1	28	2	2	3	3	10	1	2	2	2		3
	2	2	2	2	3				2	3	3	4	4
	5	3	4	3	3	5	3	5	4	1	4	3	4

	4	2	4	2	1	2	3	3	5	1	2	2	2
	2	2	2	2	2	2	1		1	1	1	1	
				2		2		2	2	2		2	
	2		2		2	2							
1	2	22	2	3	3	3	7	1	1	2	2		5
	2	2	2	2	3				2	1	1	2	4
	4	4	4	4	4	4	3	3	3	1	2	2	2
	2	1	2	1	1	2	2	2	4	2	2	2	2
				2		2		2	2	2		2	
	2		2		2	2							
1	1	20	1	2	3	3	2	1	4	2	2		5
	2	2	2	1	1	3	6	1	2	3	3	2	3
	4	2	2	4	2	3	4	5	4	1	3	4	4
	3	2	2	2	2	2	2	3	3	1	2	1	2
	1	2	1	2	2	2	2		2		1		1
		5		2		2		1	2	2		2	
	1	1	2		1	2							
1	3	21	3	2	3	3	5	3		1	2		6
	2	2	2	1	2				3	4	3	2	2
	2	2	3	1	2	3	3	3	1	3	5	4	4
	3	2	2	1	3	3	3	4	1	1	2	1	2
	1	2	1	2	1	2	3		4		6		4
		4		2				2		2			
	1	1			1								
2	1	21	1	2	3	3	2	1	1	1	2		5
	2	2	2	2	3				2	1	2	1	3
	5	3	3	4	5	5	3	3	4	2	2	2	1
	2	2	1	2	2	3	3	3	4	2	2	2	2
				2	0	2	0	2	2	2	0	2	0
	2	0	2	0	2	2							
1	1	24	2	3	3	3	8	3		1	1	1	5
	2	2	2	1	2	6	5	1	2	3	2	3	2
	3	3	2	2	3	2	2	3	2	1	2	2	3
	3	3	3	2	4	4	3	2	1	1	2	1	2
	1	2	1	2	1	2	2		2		1		1
		5		1	3	2		2	2	2		2	
	1	1	2		1	2							
2	1	23	2	2	3	3	8	1	3	2	1	1	4
	2	2	2	2	2				2	2	1	1	4
	5	3	4	3	4	5	2	4	4	1	3	3	2
	2	2	2	2	2	3	2	2	3	1	2	2	2
	2	2	2	2	2	2							
				2	0	2	0	2	2	2	0	2	0
	2	0	2	0	2	2							
2	1	23	2	2	3	3	8	1	2	2	2		4
	2	2	2	2	3				1	1	1	1	4
	5	4	5	4	4	5	3	4	4	2	1	1	1
	2	1	1	1	2	1	1	2	2	1	2	2	2
	2	2	2	2	2	2							
				2	0	2	0	2	2				
					2	2			1			1	
1	1	27	2	3	3	3	10	1	1	1	2		3
	2	2	2	2	3				2	3	3	2	3
	4	3	3	3	3	3	1	3	2	1	2		3

	3	2	2	2	2	3	2	2	2	1	2	1	2
	1	2	1	2	2	2	1		1		2		1
		1		2		2		2	2	2		2	
	2		2		2	2							
1	1	22	2	2	3	3	8	1	2	1	2		2
	2	2	2	2	3				3	2	3	3	4
	3	3	3	3	3	3	2	3	3	1	4	2	3
	3	3	2	4	2	3	4	3	3	1	2	2	2
	2	2	2	2	2	2	1		1		1		1
				2		2		2		2		2	
	2		2		2	2							
2	1	38	5	3	3	3	7	1	1	2	2		4
	2	2	2	2	2				2	2	1	2	4
	4	4	4	3	4	5	3	3	4	1	2	2	2
	2	2	2	2	2	2	2	3	3	2	2	2	2
				2		2		2	2	2		2	
	2		2		2	2							
1	1	22	2	2	3	3	2	1	2	1	2		4
	2	2	2	2	3				1	2	2	1	4
	5	5	5	4	4	3	3	4	5	2	2	1	1
	2	2	2	1	2	2	2	2	1	2	2	2	2
				2		2		2	2	2		2	
	2		2		2	2							
1	1	26	2	2	3	3	10	2	2	1	1	2	3
	2	2	2	2	3				2	2	2	2	4
	4	3	3	4	3	3	3	3	3	1	5	4	4
	3	3	2	2	1	2	4	5	1	1	2	2	2
	2	2	2	2	2	2	1		1		1		6
		6		2				2		2			
	2				2								
1	4	24	3	2	3	3	9	2	2	1	2		6
	2	2	2	1	2				3	3	2	1	4
	2	2	4	3	2	4	3	2	4	1	2	2	4
	3	2	3	2	1	2	2	4	3	1	1	1	1
	1	2	1	2	2	2	2		1		1		1
		4		2				1	2	2		2	
	1	1			1	2							
1	1	30	2	2	3	3	11	1	1	1	2		4
	2	2	2	2	3		5		2	4	3	3	4
	3	3	3	4	3	4	3	4	4	2	4	4	3
	2	2	2	3	2	3	2	2	1	1	2	1	2
	2	2	1	2	2	2	1		1		1		1
		6		2		2		2	2	2		2	
	2		2		2	2							
1	5	25	2	2	3	3	2	1	2	1	2		8
	2	2	2	2	2	6	5	1	2	1	1	2	4
	5	4	5	4	5	5	3	4	4	2	1	1	1
	2	1	2	1	1	3	2	2	4	2	2	2	2
	2												
				2	0	2	0	2	2	2	0	2	0
	2	0	2	0	2	2							
1	1	39	2	1	12	2	11	1	2	2	1	2	6
	2	2	2	2	3				1	2	2	2	4
	5	3	4	4	4	4	3	3	4	2	1	2	2

	2	1	1	2	1	2	2	1	3	1	2	2	2
	2	2	2	2	2	2							
				2		2		2	2	2		2	
	2		2		2	2							
1	3	19	1	2	3	3	2	3		2	2		4
	2	2	1	1	1	1	11	1	3	2	3	3	5
	2	3	3	3	2	3	3	5	4	2	2	4	2
	3	2	1	1	2	2	2	3	2	1	1	1	1
	1	1	1	1	1	1	3	4	2	4	6	4	4
	5	4	5	1	4	1	2	1	1	1	01	1	1
	1	1	2	0	1	1	44474	2	3	42653	3	3	
	42650	3	3										
2	1	26	3	2	3	3	5	1	1	1	2		4
	2	2	2	1	2				2	2	1	2	4
	5	3	4	3	4	5	3	4	5	3	4	1	2
	1	1	2	3	2	2	3	2	2	1	2	1	2
	1	2	2	2	2	2	1		2		1		1
		5		2		2		2	2	2		2	
	2		2		2	2							
1	1	22	3	2	2	3	6	1	1	1	2		5
	1	2	2	2	3	6	5	1	2	2	3	3	4
	4	3	3	4	3	3	2	3	3	1	4	2	4
	2	4	2	4	2	3	4	3	3	2	2	2	2
				2		2		2	2	2		2	
	2		2		2	2							
2	1	31	3	2	3	3	9	1	1	1	2		2
	2	2	2	2	3		4	1	1	1	1	1	5
	5	5	5	5	5	5	3	5	5	2	1	1	1
	1	1	1	1	1	1	1	1	4	2	2	2	2
				2		2		2	2	2		2	
	2		2		2	2							
2	1	31	2	1	12	2	11	2	2	3	2		2
	2	2	2	2	2				2	3	3	2	2
	4	2	3	3	3	3	3	3	2	2	4	4	3
	3	2	2	3	4	2	4	2	3	2	2	2	2
				2		2		1	1	2		2	
	2		2		2	2							
1	5	23	2	2	2	3	8	1	1	1	2		4
	2	2	2	1	1	3	9	2	4	3	3	3	3
	3	2	2	2	2	3	3	2	2	3	4	4	3
	3	3	4	3	3	3	3	4	1	1	1	1	2
	2	2	1	2	2	2	1		1		1		4
		5		2		2		1	1	2		2	
	2		2		2	2							
1	1	22	2	2	2	3	9	1	2	2	2		3
	2	2	2	2					1	2	2	1	4
	5	4	5	4	3	4	3	3	4	2	2	2	2
	2	2	3	2	2	3	2	2	3	2	2	2	2
				2		2		2	2	2		2	
	2		2		2	2							
2	1	20	2	2	2	3	7	1	1	2	2		2
	2	2	2	2	3	6	5	1	2	2	2	2	4
	5	5	5	3	5	5	3	5	4	2	3	2	2

	1	1	1	2	2	2	2	2	3	2	2	2	2
	2												
				2		2		2	2	2		2	
	2		2		2	2							
1	1	34	2	1	2	3	5	1	2	1	1	4	5
	2	1	1	1	1	11	12	1	2	4	4	4	3
	3	2	3	4	2	3	3	4	3	4	2	4	4
	4	4	4	4	5	3	5	2	5	1	1	1	1
	1	1	1	1	2	2	4	3	2	2	2	2	1
	1	5	5	1	3	2		2	2	2		2	
	1	3	1	1	1	1	43594	3	2	43594	3	2	
	42089	1	2										
2	1	21	2	3	2	3	1	2	1	1	2		2
	2	2	2	1	2				3	3	2	1	4
	3	2	3	3	3	4	2	3	3	1	4	3	4
	3	3	4	2	3	4	5	3	2	1	1	1	2
	2		2		2		1		2	2	3	2	6
		4		2		2		1	2	2		2	
	1	1	2		2	2							
1	1	21	2	2	2	3	5	1	3	2	2		3
	2	2	2	2	3		2	1	1	2	3	3	4
	5	3	4	3	3	3	4	4	3	2	4	3	3
	2	3	3	2	1	1	1	3	3	2	2	2	2
				2		2		2	2	2		2	
	2		2		2	2							
2	1	27	2	1	2	3	10	1	2	1	2	0	2
	2	2	2	2	3				1	1	1	2	5
	5	4	4	3	4	5	3	3	3	2	3	3	2
	2	2	1	2	4	4	2	3	3	2	2	2	2
				2	0	2		2	2	2		2	
	2		2		2	2							
1	1	23	3	2	2	3	1	1	1	2	2		3
	2	2	2	1	1	5	2	2	3	4	3	2	3
	3	3	2	3	3	1	1	3	2	1	4	3	5
	5	3	5	5	2	3	4	3	5	1	1	1	2
	1	2	1	2	1	2	4		4	1	3		4
		5		1	2	2		1	2	2		2	
	2		2		1	2							
1	1	21	2	3	2	3	9	3		2	2		4
	2	2	2	2	3				2	4	3	2	3
	3	4	2	3	2	2	2	4	2	1	3	3	3
	4	4	2	2	3	4	3	3	3	1	2	2	2
	2	2	2	2	2	2	1		1		1		4
		6		2		2		2	2	2		2	
	2		2		2	2							
2	1	25	2	2	2	3	10	1	1	1	2		5
	2	2	2	2	3				2	1	1	1	4
	5	4	4	3	4	5	3	4	4	1	1	1	2
	1	1	2	2	3	3	4	2	3	2	2	2	2
				2		2		2	2	2		2	
	2		2		2	2							
2	1	35	1	1	2	3	5	2	1	1	1	2	3
	2	2	2	2	3				2	2	1	1	4
	4	3	4	4	5	5	3	3	2	3	2	2	3

	2	3	2	3	1	3	2	2	4	2	2	2	2
				2		2		2	2	2		2	
	2		2		2	2							
2	1	19	1	2	2	3	3	1	1	3	2		6
	2	2	2	2	3				2	1	2	1	5
	5	3	3	2	3	5	3	4	5	2	3	3	3
	1	3	1	2	2	2	1	4	1	1	2	1	2
	2	2	2	2	2	2	1		2		1		1
		4		2		2		1	1	2		2	
	1	1	2		2	2							
2	3	20	2	2	2	3	3	2	5	2	2		1
	2	2	2	1	2				3	3	2		2
	1	4	4	5	2	5	2	5	5	1	2	3	2
	5	4	3	2	2	2	2	4	3	1	1	1	1
	1	1	2	2	2	2	3	2	1	1	2	2	4
	6	6	6	2		2		1	2	2		2	
	2		2		1	2							
1	1	28	2	2	2	3	5	1	1	1	2		7
	2	2	2	2	3				3	4	3	3	3
	4	3	3	3	3	3	3	3	3	3	3	3	3
	3	3	3	3	2	3	4	2	3	2	2	2	2
	2	2	2	2	2	2							
				2				2	2	2		2	
	2		2		2	2							
1	1	25	2	2	2	3	10	1	2	1	2		5
	2	2	2	2	2	6	5	1	2	2	3	2	4
	4	4	3	3	3	4	2	3	3	1	2	2	2
	2	2	2	2	3	3	4	2	3	2	2	2	2
				2	0	2	0	2	2	2	0	2	0
	2	0	2	0	2	2							
1	1	28	2	1	1	1	11	1	1	4	2		2
	2	2	2	1	2				2	4	3	3	4
	5	4	5	5	3	3	4	5	5	2	3	4	4
	3	2	1	1	1	1	1	3	5	1	2	1	2
	1	2	2	2	2	2	3	1	2		1	1	1
	1	4	6	2				2	2	2		2	
	2		2		2	2							
2	5	21	2	2	2	3	1	2	2	1	2		6
	2	2	2	2	3				2	2	1	2	4
	5	3	3	3	3	4	3	2	4	3	2	3	2
	2	3	2	3	3	3	2	2	3	2	2	2	2
				2		2		2	2	2		2	
	2		2		2	2							
2	1	22	3	2	2	3	8	3		1	2		3
	2	2	2	2	3				3	2	1	1	3
	4	4	4	3	5	4	3	4	4	2	1	2	1
	1	1	4	1	1	3	4	2	3	2	2	2	2
	2	2	2	2	2	2					1	1	6
	6	6	6	2		2		2	2	2		2	
	2		2		2	2							
2	1	20	2	2	2	3	3	2	2	1	2		3
	2	2	2	2			3	1	2	2	1	2	4
	4	4	3	3	4	5	3	3	4	1	2	1	1

	1	1	2	2	2	2	3	4	3	1	2	1	2
	2	2	1	2	2	2	1		1		1		6
		6		2		2		2	2	2		2	
	2		2		2	2							
2	1	19	3	2	2	3	2	1	1	1	2		2
	2	2	2	1	2				3	3	2	2	4
	4	3	3	3	2	4	2	3	3	2	3	3	4
	3	2	5	2	4	4	5	3	3	1	1	1	2
	1	2	2	2	2	2	1		5	5	2	2	6
	6	5	4	2		2		2	2	2		2	
	2		2		2	2							
2	1	20	1	2	2	3	7	1	1	3	2		4
	2	2	2	2					2	2	1	1	4
	4	3	4	3	4	2	2	3	5	2	3	4	2
	2	2	3	1	1	4	3	2	3	2	2	2	2
				2		2		2	2	2		2	
	2		2		2	2							
2	1	43	1	1	4	1	11	1	2	4	1	3	5
	2	2	2	1	2				1	1	3	2	4
	5	4	5	4	4	5	5	5	5	3	1	2	2
	2	3	2	2	2	3	2	3	3	1	2	2	2
	2	2	2	2	2	2	1		1		1		1
		5		2		2		2		2		2	
	2		2		2	2							
2	1	27	3	2	2	3	7	2	6	1	1	1	5
	2	1	1	1	1	12	3	2	1	1	1	1	3
	3	3	3	1	3	5	3	3	3	2	4	3	3
	2	2	2	3	3	5	1	3	3	1	2	1	2
	1	2	1	2	2	2	5		5		5		6
		4		2		2		2		2		2	
	2		2		2	2							
1	1	19	2	2	2	3	3	1	2	1	2		4
	2	2	2	2	3				2	3	3	2	3
	4	4	3	4	3	2	2	4	2	2	3	3	3
	3	4		3	3	4	3	3	3	1	2	2	2
	2	2	2	2	2	2	1		2		1	1	1
		4		2		2		2	2	2			
	2				2								
1	1	22	2	2	2	3	7	3		2	2		4
	2	2	2	1	2				2	2	1	1	2
	1	3	1	3	2	2	3	3	2	2	2	5	3
	4	3	3	3	2	2	2	5	2	1	1	1	1
	1	2	1	2	1	2	1	1	3	2	3	2	1
	1	6	6	2	0	2	0	1	2	2		2	
	2		2		2	2							
2	1	32	2	6	2	3	1	2	2	1	1	2	6
	2	2	2	1	2	6	5	1	2	1	1	3	4
	4	3	4	4	4	5	1	4	2	2	2	3	2
	1	2	2	5	2	2	5	1	2	1	2	1	2
	1	2	1	2	2	2	1		2		1		1
		4		1	1	2	0	1	2	1	1	2	0
	2	0	2	0	1	2	39479	1	3	39479	1	3	
	39479	1	3										
1	1	24	2	2	2	3	10	1	2	1	2		2
	2	1	2	2	3				2	3	2	2	4
	4	3	3	3	3	3	2	3	2	2	4	3	3

	3	3	2	4	4	4	5	4	3	2	2	2	2
				2		2		2	2	2		2	
	2		2		2	2							
2	2	37	1	2	1	1	11	3		4	2		1
	2	2	2	2	3				1	1	2	1	5
	4	3	4	5	3	4	4	4	5	2	2	1	2
	2	2	5	1	2	1	1	4	2	1	2	2	2
	2	2	2	2	2	2	1		1		1		6
		5		2		2		1	2	2		2	
	2		2		2	2							
1	1	32	2	1	1	1	11	1	2	3	1	3	5
	2	2	2	2	3				1	2	1	1	5
	5	3	4	2	4	5	5	5	3	2	3	2	2
	2	2	2	2	2	2	2	2	3	2	2	2	2
	2	2	2	2	2	2							
				2		2		2	2	2		2	
	2		2		2	2							
1	1	30	2	1	2	1	1	1	2	1	1	1	3
	2	2	1	1	1	1	3	1	2	4	1	2	2
	3	2	2	3	3	2	1	3	1	2	4	4	2
	4	2	1	3	2	4	4	4	2	1	2	1	2
	1	2	1	2	1	2	1		3		5		3
		4		2		2		2	2	2		2	
	2		2		1	2							
2	1	20	5	2	2	3	3	1	1	1	2		5
	2	2	2	2					2	1	3	2	4
	4	4	4	3	4	4	3	4	4	2	2	2	2
	2	1	2	2	2	2	2	2	4	2	2	2	2
				2		2		2	2	2		2	
	2		2		2	2							
2	1	18	2	2	2	3	1	2	1	2	2		5
	2	2	2	1	3	6	5	1	2	3	1	2	3
	4	5	3	3	4	3	4	5	2	3	2	2	1
	3	3	5	1	1	4	1	4	3	2	2	2	2
				2		2		2	2	2		2	
	2		2		2	2							
1	3	20	2	3	2	3	1	3		1	2		1
	2	2	2	1	2				3	2	2	2	1
	2	2	3	3	2	3	3	3	3	1	3	3	2
	5	3	3	5	2	3	2	3	5	1	1	1	1
	1	1	1	1	1	2	5	3	4	2	4	2	4
	6	5	6	1	1	2		1	2	2		2	
	2		1	1	1	2	44482	2	2	42607	1	2	
	42254	2	2										
2	1	21	2	3	2	3	8	1	1	2	2		4
	2	2	2	2	3				2	2	1	2	4
	5	3	4	4	3	5	2	4	4	2	2	2	1
	2	1	1	2	1	2	3	3	4	2	2	2	2
	2	2	2	2	2	2							
				2		2		2	2	2		2	
	2		2		2	2							
1	1	21	2	2	2	3	7	1	2	2	2		4
	2	2	2	2	3				3	2	2	2	2
	3	2	3	4	3	3	3	2	1	1	2	3	3

	3	4	2	3	2	2	2	2	2	1	2	1	2
	2	2	2	2	2	2	2		1		2		4
		4		2		2		1		2		2	
	2		2		2	2							
1	1	22	4	2	2	3	7	1	1	2	2		3
	2	2	2	2	3				1	1	1	1	4
	5	4	4	4	4	4	3	4	5	2	2	2	3
	2	2	2	2	1	2	1	5	2	1	2	2	2
	2	2	2	2	2	2	1		1		1		1
		6		2		2		2		2			
	2		2		2	2							
2	1	25	2	2	2	3	7	2	2	2	2		5
	2	2	2	2					2	2	2	2	3
	4	2	4	3	3	4	2	4	3	2	3	2	2
	3	2	2	3	4	3	2	2	3	2	2	1	2
	2	2	2	2	2	2	1		1		1		6
		6		2		2		2		2			
	2		2		2	2							
2	1	20	1	2	2	3	1	1	1	2	2		3
	2	2	2	2	3			1	1	1	1	2	4
	5	3	4	4	4	5	3	4	3	2	2	2	2
	1	2	2	2	1	3	2	2	3	1	2	2	2
	2	2	2	2	2	2			1		1		1
		6		2	0	2	0	2	2	2		2	
	2		2		2	2							
1	4	18	2	2	2	3	1	2	4	2	2		4
	2	2	2	1	2		13	2	3	3	3	2	2
	3	2	2	2	3	3	2	3	3	1	3	3	3
	3	4	3	3	4	4	4	3	2	1	1	1	1
	1	2	1	2	1	2	3	1	4	2	6	2	1
	1	4	4	1	10	2	0	1	1	1	11	2	0
	1	11	2	0	1	2	44491	2	2	42148	3	3	
	42148	3	3										
2	1	25	4	2	2	3	10	1	2	2	2		4
	2	2	2	2	3				2	2	1	1	4
	5	4	4	3	4	4	3	3	3	1	2	2	2
	2	3	2	2	2	3	2	2	3	2	2	2	2
	2	2	2	2	2	2							
				2		2		2	2	2		2	
	2		2		2	2							
2	1	37	1	1	2	3	5	3	1	3	1	2	3
	2	2	2	2	3				2	1	1	1	4
	5	4	4	4	5	5	5	4	4	1	2	2	2
	1	1	1	2	1	2	1	2	3	1	2	2	2
	2	2	2	2	2	2							6
	6			2		2		2	2	2		2	
	2		2		2	2							
1	1	33	2	1	2	3	10	1	2	1	1	1	3
	2	2	2	2					2	1	1		4
		2	4	2	4	4	2	4	3	2	2	2	2
	2	1	3	2	4	3	3	4	3	2	2	2	2
				2		2		2	2	2		2	
	2		2		2	2							
1	3	19	2	2	5	3	1	2	1	1	2		3
	2	2	2	1	2				3	3	3	2	3
	3	3	3	3	3	3	2	3	3	1	3	4	3

	3	3	3	4	2	3	2	3	2	1	1	1	1
	1	1	2	2	2	2	3	2	2	2	2	2	4
	4	4	4	1	1	2		1	2	2		2	
	2		2		1	2	43780	2	1		2	1	
	43780	2	1										
1	1	21	2	2	4	3	1	3		1	2		4
	2	2	2	1	1	6	5	2	4	4	3	1	1
	2	3	2	2	2	2	2	2	2	3	4	3	3
	4	5	2	5	4	3	3	3	5	1	1	1	1
	1	1	1	1	1	2	5	4	4	4	4	4	1
	6	5	5	1	1	2	1	1	1	1	1	1	
	1	5	2		1	2	44481	1	2	43780	2	3	
	41589	1	2										
1	1	22	1	2	5	3	1	1	2	1	2		4
	2	2	2	1	1	3	2	1	1	2	3	1	3
	3	2	2	4	3	3	3	4	4	1	4	4	4
	4	2	3	1	2	2	1	2	2	1	1	1	2
	1	2	1	2	1	2	4		4		4		4
		5		1	3	2		1	1	1	2	2	
	1	1			1	2	42494	1	2	42547	3	3	
	42537	3	2										
1	1	20	2	2	4	3	1	1	2	1	2		7
	2	2	2	1	2				2	1	1	1	2
	4	4	2	5	2	2	3	2	2	2	2	2	5
	3	2	1	2	1	5	2	2	2	1	2	1	2
	2	2	1	2	2	2	4		3		3		4
		5		1	3	2		1		2			
	1	3	2		1	2							
2	1	21	3	2	5	3	5	2	2	1	1	1	3
	2	2	2	2	3				2	2	1	1	4
	4	3	2	4	3	5	2	3	3	1	4	2	2
	2	2	1	2	1	2	2	1	5	2	2	2	2
	2			2									
				2		2		2	2	2		2	
	2		2		2	2							
2	1	18	2	2	2	3	1	1	2	2	2		5
	2	2	2	2	3		5	1	2	2	4	1	3
	4	4	4	3	5	5	5	3	3	2	4	4	2
	4	4	3	4	4	2	1	4	2	2	2	2	2
				2		2		2	2	2		2	
	2		2		2	2							
2	1	18	2	2	5	3	1	3	2	1	2		4
	2	2	2	2					1	1	2	1	4
	4	4	3	3	5	5	3	4	3	1	1	2	2
	1	1	2	2	1	1	1	1	3	2	2	2	2
				2		2		2	2	2		2	
	2		2		2	2							
1	1	19	2	3	4	3	3	1	1	1	2		2
	2	2	2	2	3				2	3	3	1	4
	5	2	3	3	2	3	2	3	3	3	2	3	3
	2	4	2	4	3	2	4	3	2	1	2	1	2
	2	2	2	2	2	2	1		1		4		1
		4		2		2		1	2	2		2	
	2		2		2	2							
1	1	20	4	1	4	3	3	3		1	2		2
	2	2	2	2	3				2	2	1	1	4
	4	2	4	3	3	5	1	1	2	1	2	3	3

	2	4	2	4	4	4	5	2	2	2	2	2	2
	2	2	2	2	2	2							
				2		2		2	2	2		2	
	2		2		2	2							
1	1	18	4	2	5	3	1	1	1	1	2		6
	2	2	2	2	3				2	1	1	2	4
	4	4	3	4	4	4	2	3	4	2	2	3	2
	2	2	1	2	2	3	3	2	3	2	2	2	2
				2		2		2	2	2		2	
			2		2	2							
1	1	19	2	3	5	3	1	1	2	1	2		8
	2	2	2	1	2				2	2			3
		2	3	3	3	3	3	3	3	1	2	2	2
	3	3	2	3	2	2	3	2	3	1	2	1	2
	1	2	1	2	2	2	4		1		2		1
		5		2		2		2		2			
					1	2							
2	1	18	2	2	5	3	1	1	1	2	2		4
	2	2	2	2	3	6	5	1	2	2	2	2	4
	4	3	3	3	3	3	3	5	4	1	3	4	4
	2	2	2	3	1	2	2	2	3	2	2	2	2
	2	2	2	2	2	2	1	1	1	1	3	3	6
	6	6	6	2	0	2	0	1	2	2	0	2	0
	2	0	2	0	2	2		1	1		1	1	
	1	1											
1	2	19	2	2	5	3	1	2	1	1	2		3
	2	2	2	2	3				3	3	3	3	3
	3	3	2	2	3	3	3	3	3	3	2	3	3
	3	3	3	2	2	3	4	2	1	1	2	1	2
	1	2	1	2	2	2	1		1		1		6
		5		2		2		1	2	2		2	
	2		2		1	2							
1	1	26	2	1	2	3	10	1	2	1	2	2	2
	2	1	2	2	3				2	2	3	3	3
	3	2	2	4	3	4	4	4	2	2	2	2	2
	2	2	2	2	2	2	2	2	2	1	1	2	2
	2	2	2	2	2	2	5	1	1	1	3	3	6
	6	4	4	2		2		2	2	2		2	
	2		2		2	2							
2	1	23	2	2	9	3	8	1	1	1	2		3
	2	1	2	2	2				2	2	3	4	3
	5	4	3	3	3	5	2	4	4	2	1	2	2
	2	1	3	1	1	3	3	2	2	2	2	1	2
	2	2	2	2	2	2	1		2		2		1
		6		2		2		1	2	2		2	
	2		2		2	2							
1	1	21	2	1	5	3	1	1	2	1	2		7
	2	2	1	2					2	2	3	4	2
	3	2	2	3	3	3	2	3	2	2	4	4	4
	4	3	2	2	2	2	4	2	5	1	2	1	2
	2	2	2	2	2	2					4		4
		5	5	2		2		1	1	2		2	
	2		2		2	2							
1	1	22	2	2	5	3	1	1	1	1	2		9
	2	2	2	1	2	6	5	2	2	2	1	1	3
	3	3	3	4	3	5	3	3	2	1	2	3	2

	2	2	3	1	2	4	2	2	3	1	2	1	2
	1	2	1	2	1	2	4		4		5		3
		4		1	1		0	2	2	2	0	2	0
	1	1	2	0	1	2			3			1	
	5	3											
2	1	19	1	2	4	3	1	1	2	2	2		4
	2	2	2	2	3				2	3	2	1	3
	4	2	2	4	4	5	2	5	4	1	4	4	4
	4	2	3	2	2	3	3	3	3	2	2	2	2
				2		2		2	2	2		2	
	2		2		2	2							
2	1	19	2	2	4	3	1	1	2	1	2		4
	2	2	2	1	2				2	2	1	1	2
	3	3	3	4	4	3	2	4	4	3	1	3	3
	3	2	1	3	2	2	4	3	3	1	2	1	2
	2	2	2	2	2	2	3		2		6		6
		6		2		2		2	2	2			
	2				2	2							
1	1	23	3	3	5	3	8	1	3	1	2		2
	2	2	2	1	1	5	6	2	3	4	4	3	2
	2	1	2	3	1	3	2	4	3	2	5	4	4
	5	5	4	4	3	3	4	3	5	1	1	1	2
	1	1	1	2	2	2	5	5	3	3	4	4	1
	1	5	5	2		2		2	2	2		2	
	2		2		2	2							
2	1	27	3	2	5	3	8	1	3	1	2		1
	2	1	2	1	2				2	2	1	1	3
	4	4	4	4	4	4	2	3	3	2	1	2	2
	2	4	1	2	2	4	4	2	3	1	1	1	2
		2	1	2	2	2	4		2		4		1
		5		2				2					
2	2	36	3	3	1	1	11	1	5	3	2		3
	2	2	2	2	3				2	2	2	2	3
	3	3	3	3	3	4	4	4	3	1	2	2	2
	2	2	2	3	2	3	3	3	3	1	2	2	2
	2	2	2	2	2	2	1		1		1		6
		6		2		2		2	2	2		2	
	2		2		2	2							
1	1	19	2	2	10	3	7	1	2	2	2		6
	2	2	2	1	1	3	2	1	2	3	2	2	3
	4	3	3	3	3	2	2	3	2	1	3	3	2
	5	4	3	4	4	4	4	4	2	1	1	1	2
	1		1	2	1	2	1		4		5		4
		4		1	3	2		1	2	1	1	2	
	1	1	2		1	2	44459	1	3	44459	1	3	
	44599	1	2										
1	1	28	3	3	10	3	6	1	1	1	2		6
	2	2	2	2	3				2	2	1	3	4
	5	3	3	3	3	4	2	4	4	2	2	2	2
	2	2	1	2	2	3	4	2	3	2	2	2	2
	2	2	2	2	2	2	1	1	1	1	1	1	1
	1	6	6	2		2		2	2	2		2	
	2		2		2	2							
2	1	29	3	4	10	3	8	3		1	1	1	2
	2	2	2	2	3				4	1	1	1	4
	2	5	3	2	5	5	2	4	4	2	3	2	2

	2	2	4	2	1	3	3	3	3	2	2	2	2
				2		2		2	2	2		2	
	2		2		2	2							
2	1	36	2	2	10	3	7	1	2	2	2		5
	2	2	2	2	3	6	5	1	1	2	2	2	4
	4	3	4	3	5	5	3	4	3	1	3	2	2
	2	2	3	2	2	4	4	5	3	2	2	2	2
				2		2		2	2	2		2	
	2		2		2	2							
1	1	19	2	2	10	3	1	1	1	1	2		3
	2	2	2	2	2				3	2	4	1	2
	5	3	2	4	2	3	2	4	2	3	5	3	3
	1	3	4	1	3	2	5	2	1	1	1	2	2
	2	2	1	2									
		4		2		2		2	2	2		2	
	1	1	2		2	2							
1	1	22	3	1	10	3	6	1	2	1	2		10
	2	2	2	2	3				2	2	1	2	4
	5	4	5	4	5	5	3	3	3	1	3	2	2
	1	1	1	3	2	3	3	2	3	2	2	2	2
				2		2		2	2	2		2	
	2		2		2	2							
1	1	19	2	1	10	3	2	1	1	2	2		3
	2	2	2	2	3			2	1	2	2	2	5
	5	4	2	3	5	3	3	3	3	2	2	2	2
	1	1	1	2	2	3	2	1	3	2	2	1	2
	2	2	2	2	2	2	1		1		1		6
		6		2	0	2	0	2	2	2	0	2	0
	2	0	2	0	2	2							
2	1	33	2	1	10	3	3	1	1	3	1	2	4
	2	2	2	2	3				1	2	1	1	3
	5	4	4	3	4	4	4	4	4	2	2	2	2
	2	2	2	2	2	4	2	2	3	2	2	2	2
				2	0	2	0	2	2	2	0	2	0
	2	0	2	0	2	2							
2	1	20	2	2	10	3	4	2	1	1	1	2	4
	2	2	2	1	1	6	14	1	1	3	2	2	4
	5	4	4	3	3	5	3	3	4	1	3	2	2
	2	3	3	3	2	2	2	1	1	1	2	1	2
	1	2	1	2	1	2	4		1		3		1
		6		2		2		1	2	2		2	
	1	1	2		1	2							
1	1	23	3	2	10	3	6	1	1	1	2		3
	2	2	2	2	3				2	3	5	3	4
	4	3	1	3	3	2	1	4	4	1	4	2	2
	4	5	3	4	3	4	2	3	2	1	2	2	2
	2	2	2	2	2	2	1		1		3		1
		4		2		2		2	2	2		2	
	2		2		2	2							
2	1	47	2	1	8	1	11	2	1	3	1	2	3
	2	1	2	1	2				3	4	2	3	4
	4	3	4	3	4	4	3	4	4	1	3	2	2

	2	2	2	3	2	4	2	2	3	2	2	2	2
				2	2	2	0	2	2	2	0	2	0
	2	0	2	0	2	2							
1	1	57	1	1	7	1	11	1	1	3	1	4	3
	1	2	2	2					2	4	2	3	3
	4	4	4	4	4	4	3	4	3	2	2	2	2
	1	1	2	1	2	2	1	2	3	2	2	2	2
				2		2		2	2	2		2	
	2		2		2	2							
1	1	31	2	2	10	3	7	1	2	1	1	2	3
	2	2	2	2	3				3	2	1	1	4
	4	3	3	3	4	5	2	3	3	1	3	3	3
	3	2	3	3	4	3	4	3	3	2	2	2	2
	2												
				2		2		2	2	2		2	
	2		2		2	2							
1	1	23	2	2	10	3	4	2	2	1	1	1	5
	2	2	2	2	3				2	2	3	1	4
	3	4	4	4	4	3	3	4	2	1	2	2	2
	2	2	2	2	2	3	3	2	3	2	2	2	2
				2		2		2	2	2		2	
	2		2		2								
1	1	26	2	3	10	3	8	2	1	2	2		2
	2	2	2	2	3				2	5	1	2	4
	4	3	3	3	4	4	2	4	3	1	2	2	4
	2	2	2	3	2	2	4	2	3	2	2	1	2
	2	2	2	2	2	2	1		1		1		6
		1		2		2		2		2		2	
	2		2		2	2							
2	1	25	2	2	10	3	7	1	3	1	2		5
	2	2	2	1	2				2	2	2	2	2
	5	3	4	2	4	4	3	2	2	4	4	4	4
	4	2	2	2	4	4	2	4	2	1	2	1	2
	1	2	1	2	2	2	4		5		5		4
		4		2		2		2	2	1		2	
	2		2		1	2							
1	1	24	2	3	10	3	5	2	1	1	1	2	7
	2	2	2	1	2				3	3	2	2	2
	3	3	2	2	2	3	2	2	1	3	4	4	3
	3	3	4	3	4	4	5	2	1	1	2	1	2
	1	2	1	2	2	2	1		2		3		4
		5		2		2		2	2	1	6	2	
	2		2		2	2							
1	1	22	2	2	10	3	8	2	2	2	2		6
	2	2	2	2	3				3	5	3	3	2
	4	3	3	4	3	3	4	4	2	1	4	4	4
	4	3	3	3	2	3	2	3	5	1	2	2	2
	2	2	2	2	2	2	1		1		1		6
		6		2		2		2		2			
	2				2								
2	1	31	3	2	10	3	5	3		1	1	1	1
	2	2	2	1	2				4	3	1	1	2
		3	2	3	5	5	2	3	2	1	2	3	2

	4	2	3	2	4	4	3	3	2	1	2	1	2
	2	2	1	2	2	2	4		4		1		4
		4		2		2		2	2	2		2	
	1	1	2		2	2							
1	1	22	2	1	10	3	8	1	1	1	2		2
	2	2	2	2	3				3	3	1	2	3
	3	2	2	3	3	3	2	3	2	1	2	3	2
	3	3	3	3	3	3	4	3	2	1	1	1	1
	1	2	1	2	1	2	2		2		2		1
		4		2	0	2	0	2	2	2	0	2	0
	2	0	2	0	2	2							
1	1	26	1	1	10	3	5	1	2	1	2		6
	2	2	2	2	3				2	3	3	1	4
	5	4	4	3	3	4	3	4	4	2	3	2	2
	3	1	2	1	1	3	1	2	3	2	2	2	2
				2		2		2	2	2		2	
	2		2		2	2							
2	1	35	1	1	12	3	11	1	2	2	1	2	4
	2	2	2	1	1	3	6	1	2	3	1	1	4
	5	4	4	4	4	3	3	4	3	1	2	2	2
	2	2	2	2	2	3	2	3	4	2	2	1	2
	2	2	2	2	2	2	1		1		1		1
		1		1	2	2		1	2	1	1	2	
	1				1	2							
1	1	19	2	2	10	3	1	1	1	1	2		3
	2	2	2	2	3				2	3	3	2	4
	4	4	3	4	4	3	2	3	4	2	3	4	4
	2	3	4	2	2	2	4	2	1	1	1	2	2
	2	2	2	2	2	2	2	1	1	1	1	1	1
	1	5	5	2		2		2	2	2		2	
	2		2		2	2							
2	2	19	2	2	10	3	1	2	1	1	2		6
	2	2	2	1	1	3	6	1	3	3	3	2	3
	1	2	2	1	3	3	3	3	2	1	4	3	4
	4	3	4	3	2	3	3	3	5	1	1	1	1
	1	1	1	1	1	2	4	3	2	1	3	3	3
	4	5	4	1	2	1	1	1	2	1	1	2	0
	2		1	1	1	1	44534	2	2	44534	2	2	
	43830	2	2										
2	1	23	2	2	10	3	4	1	2	3	2		8
	2	2	2	2	3				3	3	1	3	4
	4	3	3	4	4	3	3	4	4	2	4	2	2
	3	3	3	2	2	3	5	2	3	2	1	1	1
				2	0	2		2	2	2		2	
	2		2		2	2							
2	1	31	2	2	10	3	7	3		3	2		4
	2	2	2	2	3				3	4	3	3	3
	5	2	3	3	3	5	3	4	3	1	4	2	3
	2	2	2	3	2	3	3	4	1	2	2	1	2
	2	2	2	2	2	2	1		1		3		6
		6		2		2		2		2		2	
	2		2		2	2							
1	1	28	2	2	10	3	8	1	2	1	1	3	5
	2	2	2	2	3				1	4	4	3	4
	4	2	4	2	3	5	3	3	2	1	5	3	2

	1	1	1	1	3	5	1	2	2	1	2	2	2
	2	2	2	2	2	2	5		1		1	1	1
	1	5	5	2		2		2	2	2		2	
	2		2		2	2							
1	1	20	2	3	10	3	4	1	2	2	2		6
	2	2	2	1	1	3	2	1	1	3	4	3	5
	4	5	3	3	3	3	3	5	5	2	2	5	5
	3	2	3	3	1	1	1	3	2	1	2	1	2
	1	2	2	2	2	2	1		2		3		1
		5		1	1	2		2	2	2		2	
	2		2		1	2	43288	3	3	43288	3	3	
	43288	3	3										
2	1	18	2	2	10	3	2	1	2	1	2		5
	2	2	2	2	3				1	1	1	1	5
	5	5	5	5	5	5	3	4	4	2	1	1	1
	1	1	1	2	2	2	1	1	4	2	2	2	
				2	0	2	0	2	2	2	0	2	0
	2												
1	1	42	5	1	11	3	8	1	2	1	1	3	4
	2	2	2	1	1	6	5	1	2	4	3	3	4
	4	3	4	4	3	3	3	3	3	1	4	4	3
	3	2	3	3	2	4	4	3	3	1	2	1	2
	1	2	1	2	1	2	5		5		4		1
		5		1	1	2		2	2	2		2	
	2				1	2		4	2				
1	1	21	2	3	9	3	8	1	4	2	2		2
	2	2	1	1	1	8	13	2	2	3	3	1	4
	5	4	4	4	3	3	4	5	4	1	3	2	2
	3	2	1	1	1	2	2	4	3	1	2	1	2
	1	2	1	2	1	2	3		3		5	1	1
		4		1	1	2	0	2	2	2	0	2	0
	1	1	2	0	1	2	43585	3	2	43585	3	2	
	43585	3	2										
1	3	47	1	4	9	3	8	1	8	3	1	3	3
	1	2	1	1	1	3	4	1	2	3		5	4
	4	3	3	3	3	4	4	3	3	4	1	4	5
	1	3	4	4	5	5	3	4	3	1	2	1	2
	2	2	1	2	2	2	3		1		2		1
		4		2	0	2	0	2	2	2	0	2	0
	2	0	2	0	2	2							
2	1	35	2	2	9	2	3	1	1	2	2		1
	2	2	2	2	3				2	2	2	3	4
	4	4	3	3	3	5	3	4	3	1	3	2	2
	2	2	2	2	4	3	2	2	2	2	2	2	2
				2		2		2	2	2		2	
	2		2		2	2							
1	1	23	2	1	9	3	8	1	2	2	2		3
	2	2	2	2	3				1	2	2	2	4
	5	4	5	4	4	5	4	4	4	2	1	2	1
	2	2	1	2	2	2	1	3	3	2	2	2	2
				2		2		2	2	2		2	
	2		2		2	2							
1	1	21	2	2	9	3	7	1	1	2	2		4
	2	1	2	2	3				2	3	2	1	3
	4	3	4	4	3	5	3	4	3	1	4	3	3

	2	2	2	2	1	2	2	3	3	1	2	2	2
	2	2	2	2	2	2	1		2				
				2		2		2	2	2		2	
	2		2		2	2							
1	1	24	2	1	9	3	6	1	2	3	1	1	3
	2	2	2	2	2				1	2	2	1	4
	4	3	4	3	3	3	4	4	3	1	2	2	2
	2	2	2	2	2	3	2	2	3	1			
	2	2	2	2	2	2	1		1		1		1
		4		2		2		2	2	2		2	
	2		2		2	2							
1	1	22	2	1	9	3	8	1	1	2	1	1	3
	2	2	2	2	3				2	3	2	1	4
	4	4	4	3	3	3	3	5	4	2	2	2	2
	3	3	2	3	2	3	2	2	2	2	2	2	2
				2		2		2	2	2		2	
	2		2		2	2							
1	1	23	2	1	9	3	8	3		2	1	1	3
	2	1	2	1	1	13	2	1	2	2	4	2	3
	3	2	2	4	2	3	4	5	3	2	4	4	3
	3	3	2	3	3	2	2	4	5	1	1	1	2
	1	2	1	2	2	2	2		2		3		1
		4		2		2		1	2	2		2	
	1	3	2		2	2							
2	1	27	3	2	9	3	1	1	2	3	2		7
	2	2	2	2	3				2	2	2	1	3
	5	2	3	4	3	4	3	4	4	2	4	4	2
	3	2	2	1	2	4	2	5	3	1	2	1	2
	2	2	2	2	2	2	1		1		1		1
		4		2		2		2	2	2		2	
	2		2		2	2							
1	1	23	5	2	9	3	8	1	7	2	2		3
	2	2	2	1	2				2	4	2	2	4
	4	3	3	4	3	4	2	3	2	1	2	3	3
	4	3	4	2	1	2	4	4	2	1	2	1	2
	1	2	1	2	2	2	3		2		1		3
		5		1		2		1	2	2		2	
	1		2		1	2							
2	1	33	2	1	9	3	5	1	2	2	2		2
	2	2	2	2					2	4	1	1	4
	5	3	4	4	3	4	3	4	2	2	4	3	2
	2	2	2	3	2	4	2	2	3	2	2	2	2
				2	1	2	1	2	2	2	1	2	1
	2	1	2	1	2	2							
1	1	21	2	2	9	3	8	3		2	2		1
	2	2	2	1	2				2	2	1	1	3
	4	2	4	4	3	2	3	4	2	2	2	2	2
	3	3	2	1	2	2	4	5	2	1	2	1	2
	1	2	2	2	2	2	1		1		2		4
		5		2		2		2	2	2		2	
	2		2		2	2							
2	1	22	1	3	9	3	7	2	1	3	2		3
	2	1	2	1	2				1	4	2	1	3
	4	2	4	4	5	2	5	4	3	2	4	5	5

	4	2	1	2	1	2	5	5	1				
	1	2	2	2	1	2	3		2		3	2	6
		6		2		2		1	2	2		2	
	2				1	2							
1	1	29	2	1	9	3	8	1	1	2	1	2	3
	2	2	2	2	2				3	3	3	2	2
	4	2	4	4	3	4	3	3	2	1	2	3	3
	2	4	4	2	2	4	4	2	3	1	2	2	2
	2	2	2	2	2	2	2		1		1		3
		6		2	0	2	0	2	2	2	0	2	0
	2		2		2	2							
2	1	22	2	2	9	3	7	1	1	1	2		4
	2	2	2	2	3				2	1	1	1	4
	4	3	4	3	5	4	3	3	2	1	2	2	2
	2	2	2	1	2	2	4	1	3	2	2	2	2
				2		2		2	2	2		2	
	2		2		2	2							
2	1	28	2	2	9	3	1	1	1	3	2		3
	2	2	2	2					2	2	2	1	4
	4	3	4	4	5	5	5	4	4	2	1	1	1
	1	2	2	2	2	2	2	2	3	2	2	2	2
				2		2		2	2	2		2	
	2		2		2	2							
2	1	21	2	3	9	3	8	2	1	3	2		4
	2	2	2	2	3				1	2	3	1	4
	5	3	4	3	4	5	4	4	4	2	4	4	3
	2	1	1	1	1	3	2	2	3	2	2	2	2
				2		2		2	2	2		2	
	2		2		2	2							
1	1	23	2	2	9	3	5	2	1	1	2		2
	2	2	2	2	3				3	2	2	3	3
	4	3	3	3	3	3	2	4	3	1	3	4	3
	3	3	2	3	2	3	3	2	3	1	2	1	2
	1	2	1	2	2	2	1		1		2		1
		3		2		2		2	2	2		2	
	2		2		2	2							
2	1	24	1	2	9	3	6	1	3	2	2		6
	2	2	2	2	3				2	2	2	2	3
	4	4	3	4	4	5	2	5	2	2	2	2	2
	2	4	2	1	4	5	1	4	3	2	2	2	2
				2	0	2	0	2	2	2	0	2	0
	2	0	2	0	2	2							
2	1	22	4	2	9	3	5	1	2	1	2		5
	2	2	2	2	3				2	1	1	1	4
	5	4	4	4	3	5	4	5	4	1	3	2	2
	1	2	2	2	1	2	2	1	3	2	2	2	2
				2		2		2	2	2		2	
	2		2		2	2		2					
2	1	21	1	3	9	3	5	1	1	2	2		5
	2	2	2	2	3				1	1	1	1	5
	5	4	4	4	5	5	3	4	5	2	3	1	1

	1	1	1	1	2	2	1	3	3	1	2	2	2
	2	2	2	2	2	2	1		1		1		6
		4		2		2		1	2	2		2	
	1	1	2		2	2							
1	3	19	2	3	9	3	4	3		2	2		6
	2	2	2	2	3				3	5	3	4	4
	4	3	3	2	3	3	4	5	2	3	4	2	1
	2	3	2	3	4	3	4	3	1	1	1	1	2
	1	2	1	2	2	2	2		2		2		5
	6	4		2	0	2	0	1	2	2	0	2	0
	2	0	2	0	1	2							
2	1	24	2	2	9	3	2	1	2	1	2		3
	2	2	2	1	1	6	5	1	3	3	4	1	2
	4	2	3	4	4	5	1	4	5	2	4	3	4
	3	2	4	2	2	2	4	3	3	1	2	1	2
	1	2	2	2	2	2	1		2		2	2	1
		6		2		2		2	2	2		2	
	2		2		2	2							
2	1	20	3	2	9	3	2	1	1	3	2		4
	2	2	2	2	3	6	5		1	5	1	1	5
	5	4	5	5	5	5	5	5	5	2	1	1	1
	1	1	1	2	1	3	1	1	4	2	2	2	2
	2	2	2	2	2	2							
				2				2	2	2		2	
	2		2		2	2							
1	1	22	2	1	9	3	6	1	2	2	2		2
	2	2	2	1	2				2	2	4	2	3
	4	3	3	4	3	4	3	4	4	1	4	4	4
	4	4	4	4	4	4	4	4	2	1	1	1	2
	2	2	2	2	2	2	1		1		1		6
	6	3		2		2		2	2	2		2	
	2		2		2	2							
2	1	19	1	2	9	3	1	1	3	3	2		5
	2	2	2	2	3				2	2	1	1	4
	4	4	4	4	5	5	2	4	3	2	1	1	2
	2	2	2	3	1	2	1	2	3	2	2	2	2
				2		2		2	2	2		2	
	2		2		2	2							
2	1	19	2	2	9	3	3	1	2	3	2		5
	2	2	2	2	3				2	3	2	1	4
	5	4	4	3	3	5	5	5	3	1	3	2	2
	2	3	1	3	1	3	1	3	3	2	2	2	2
				2		2		2	2	2		2	
	2		2		2	2							
1	1	21	2	3	9	3	3	1	3	1	2		5
	2	2	2	1	2				3	3	1	1	2
	3	2	2	1	2	3	2	4	2	1	2	3	3
	5	2	2	1	5	3	3	2	3	1	2	1	2
	1	2	1	2	1	2	2		1		1		1
		4		2		2		1	2	2		2	
	2		2		1	2							
1	1	22	2	2	9	3	8	2	1	2	2		5
	2	2	2	2	3				2	2	3	1	3
	4	3	3	3	3	3	3	4	3	1	2	3	3

	4	2	1	2	3	3	2	2	3	2	2	2	2
				2		2		2	2	2		2	
	2		2		2	2							
1	3	21	2	2	9	3	4	2	3	1	2		6
	2	2	2	1	1	1	14	1	2	4	3	1	4
	4	3	3	1	4	2	2	3	2	5	3	2	2
	2	4	3	4	4	4	5	4	2	1	2	1	2
	1	2	1	2	1	2	4		4		6		1
		5		1	3	2		1	2	1		2	
	1		2		1	2	43018	1	3	43018	1	2	
	43018	1	2										
1	1	19	2	2	9	3	4	2	1	2	2		3
	2	2	2	2	3				2	2	3	2	4
	4	3	3	3	3	3	2	4	3	1	2	3	3
	2	3	4	4	3	4	2	3	3	2	2	2	2
				2		2		2	2	2		2	
	2		2		2	2							
1	1	27	2	2	9	3	3	1	1	1	2		1
	2	2	2	2	3				2	3	3	3	2
	3	3	3	4	3	4	2	3	3	1	3	3	3
	2	2	3	3	2	4	2	2	3	2	2	2	2
				2		2		2	2	2		2	
	2		2		2	2							
1	3	18	3	2	9	3	2	2	1	3	2		4
	2	2	1	1	1	14	13	1	2	4	3	4	2
	2	3	4	3	2	3	3	4	4	1	3	3	4
	4	3	3	2	3	3	1	3	5	1	1	1	1
	1	1	1	1	1	1	4	1	4	2	5	2	1
	1	5	4	2		2		1	1	2		2	
	1	1	2		1	1							
2	5	19	2	2	9	3	2	2	1	1	2		4
	2	2	2	2	3	6	5	2	2	3	3	3	3
	4	4	4	3	4	3	3	3	3	1	3	4	3
	2	1	3	2	2	2	2	2	2	2	2	2	2
				2	0	2	0	2	2	2	0	2	0
	2	0	2	0	2	2							
2	2	25	4	2	9	3	4	2	3	2	2		3
	2	2	2	1	2				2	3	1	2	2
	3	4	4	3	2	3	2	4	3	1	2	4	5
	4	4	5	3	3	4	4	4	2	1	1	2	2
	2	2	1	2	2	2					1	1	6
	6	6	6	2		2		2	2	2		2	
	2		2		2	2							
2	1	19	3	2	9	3	3	1	1	2	2		4
	2	2	2	2	3				3	3	3	1	3
	2	3	3	5	3	3	3	2	4	2	4	3	2
	3	4	3	3	3	4	4	3	3	1	1	1	2
	1	2	1	2	2	2			2		6		1
		6		2		2		2	2	2		2	
	1				2	2							
1	4	24	3	3	9	3	4	2	1	1	2		6
	2	2	2	2	3				2	4	3	3	3
	2	2	3	3	3	3	2	4	2	2	4	3	3

	4	3	2	2	4	3	4	3	2	1	1	1	1
	1	2	1	2	2	2	1	1	1	1	2	1	1
	1	4	4	2		2		1	2	2		2	
	2		2		1	2							
2	1	24	5	3	9	3	4	1	2	1	2		4
	2	2	2	2	3				2	2	3	3	3
	4	3	3	4	3	5	2	3	2	1	3	2	2
	2	2	2	3	2	3	1	2	3	2	2	2	2
				2		2		2	2	2		2	
	2		2		2	2							
2	1	44	2	1	8	3	8	1	1	1	1	3	5
	2	2	2	1	2				2	2	2	2	3
	4	4	4	4	4	4	3	3	3	2	2	2	2
	2	2	2	2	2	3	2	2	3	1	2	1	2
	1	2	1	2	1	2			1		2	1	1
	6	4		2		2		2	2	2		2	
	2		2		2	2							
2	1	22	2	3	8	3	8	1	3	1	2		4
	2	2	2	2	3				2	2	2	1	3
	4	3	3	3	3	5	3	3	2	1	4	3	3
	2	2	2	4	2	2	2	2	3	2	2	2	2
				2		2		2	2	2		2	
	2		2		2	2							
1	1	22	2	3	8	3	8	1	1	2	2		4
	2	2	2	1	2				2	2	1	1	4
	5	3	3	5	3	3	3	5	4	2	1	2	2
	2	1	1	2	2	2	3	1	3	2	2	1	2
	2	2	2	2	2	2	1		2		1		1
		5		2		2		1	2	2		2	
	2		2		2	2							
2	1	22	2	2	9	3	1	1	2	1	2		5
	1	2	2	2	3				2	2	2	3	4
	4	4	5	4	5	5	3	4	3	4	2	2	2
	2	2	2	2	2	4	4	2	3	2	2	2	2
	2												
				2		2		2	2	2		2	
	2		2		2	2							
1	1	19	2	3	9	3	2	2	1	1	2		5
	2	2	2	2	3				3	4	3	3	3
	4	4	2	2	3	2	2	3	2	3	4	3	3
	4	2	2	3	4	4	3	3	1	1	1	1	2
	2	2	1	2	2	2	1		3		3		4
		6		2		2		2	2	2		2	
	2		2		1	2							
2	1	29	3	2	9	3	2	2	1	1	2		3
	2	2	2	2	3	6	5	2	2	2	2	1	4
	5	3	4	4	5	5	3	3	2	2	1	1	1
	1	1	1	2	1	4	1	2	3	2	2	2	2
				2		2		2	2	2		2	
	2		2		2	2							
1	5	26	2	2	8	3	8	2	1	1	2		3
	2	2	2	2	3				1	1	1	1	5
	4	4	5	4	5	5	3	4	4	2	2	1	1

	1	2	1	2	2	2	2	3	3	2	2	2	2
				2		2		2	2	2		2	
	2		2		2	2							
1	1	21	2	2	8	3	8	1	2	2	2		5
	2	2	2	2	3				2	3	2	1	4
	5	3	3	3	3	3	3	4	4	2	2	3	2
	2	2	1	2	2	2	2	2	2	2	2	2	2
	2	2	2	2	2	2	1						
				2		2		2	2	2		2	
	2		2		2	2							
1	1	20	2	2	6	3	4	1	1	3	2		5
	2	2	2	2	3				2	2	1	1	4
	5	4	4	4	4	4	4	4	3	2	2	2	2
	2	1	1	3	3	2	1	2	3	2	2	2	2
				2		2		2	2	2		2	
	2		2		2	2							
1	1	19	2	2	9	3	1	1	2	1	2		5
	2	2	2	2	2	6	5	2	3	2	1	1	3
	3	2	3	3	2	3	2	2	2	5	4	3	3
	3	3	3	3	3	3	3	3	3	2	2	2	2
				2		2		2	2	2		2	
	2		2		2	2							
1	1	23	2	2	8	3	8	1	2	1	2		3
	2	2	2	2	3				2	3	3	2	3
	4	3	4	4	3	2	3	4	2	3	4	2	2
	4	2	4	2	3	2	4	2	3	1	1	2	2
	1	2	2	2	2	2	1		2		1		1
	1	4	4	2		2		2	2	2		2	
	2		2		2	2							
1	4	23	2	4	8	3	8	3		1	2		5
	2	2	2	1	1	3	2	1	2	4	3	4	3
	2	2	2	3	2	1	2	3	3	1	5	4	3
	5	4	5	2	4	3	5	3	1	1	1	1	1
	1	1	1	1	1	2	5	3	3	1	4	2	4
	1	5	6	1	5	1	1	1	1	1	5	2	
	2		1	1	1	2	44545	1	3	43758	3	2	
	43537	1	3										
2	1	33	2	2	8	3	8	1	1	2	2		2
	2	2	22	1	1	3	2	1	2	2	1	2	3
	4	3	2	4	3	3	3	3	3	1	2	3	4
	4	2	4	2	5	4	4	4	2	1	1	1	1
	1	1	1	2	1	2	1	1	1	1	1	1	1
	1	3	3	2		2		2	2	2		2	
	2		2		1	2							
1	1	21	2	2	8	3	8	1	1	2	2		5
	2	2	2	1	1	2	6	1	1	5	3	2	3
	5	4	4	4	4	4	3	4	3	2	3	1	1
	1	2	3	2	1	3	1	2	3	1	2	1	2
	1	2	2	2	2	2	4				5		1
		4		2				2	2	2		2	
	2		2		2	2							
1	4	19	3	2	9	3	2	3		1	2		3
	2	2	2	1	2				1	2	1	1	3
	3	3	3	5	3	5	3	4	3	2	3	2	2

	1	1	2	2	1	2	3	2	1	1	2	1	2
	1	2	1	2	1	2	3		3		1		4
		5		1	1	2		1		1	1	2	
	1	1	2		1	2	42855	2	3	42855	2	3	
	42855	2	3										
1	1	19	2	3	9	3	2	3		1	2		4
	2	2	2	2	3				3	3	3	2	3
	2	2	1	1	3	2	1	3	2	3	5	3	2
	4	3	2	4	4	3	2	2	5	1	1	1	1
	2	2	1	1	2	2	4	4	2	5	3	4	1
	1	4	4	2		2		2	2	2		2	
	2		2		2	2							
1	1	25	2	2	8	3	8	1	2	1	2		4
	2	2	2	2	3				2	2	2	2	4
	5	4	4	4	4	4	3	4	4	1	2	2	2
	1	1	3	2	3	3	3	3	3	2	2	2	2
				2		2		2	1	1		2	
	2		2		2	2							
1	1	23	3	2	8	3	8	3		1	2		4
	2	1	1	1	1	8	4	1	2	3	2	2	3
	4	4	4	2	3	4	2	3	3	1	3	2	2
	2	2	2	2	2	4	2	4	3	1	2	1	2
	1	2	2	2	2	2	5		5		6		1
		5		2		2		1	2	2		2	
	2		2		1	2							
2	1	23	4	2	8	3	6	1	2	1	2		3
	2	2	2	2	3				2	2	2	2	4
	3	3	3	4	4	4	1	4	4	1	2	2	2
	2	3	3	3	2	4	4	2	3	1	2	1	2
	1	2	2	2	2	2	1		3		1		1
		4		2		2		2					
					2								
2	1	20	2	2	9	3	2	1	3	1	2		5
	2	2	2	1	2				3	2	1	1	3
	4	3	2	2	3	3	2	3	3	1	4	4	4
	5	4	5	2	4	3	4	3	3	2	1	1	2
	2	2	1	2	2	2	1		2		3		1
		5		2		1		2	2	2		2	
	1	7	2		2	2							
1	5	22	2	2	8	3	8	2	2	2	2		5
	2	1	2	1	2				3	4	1	2	3
	3	3	3	3	3	3	3	4	3	2	3	4	4
	4	4	4	3	3	3	3	3	3	1		1	
	1		1		2		1		2		3		4
		4	4	2		2		1	1	2		2	
	2		2		2	2							
1	1	24	2	2	8	3	8	2	2	1	1	1	7
	2	2	2	1	2				2	1	1	2	3
	4	3	3	3	3	3	2	3	3	1	3	3	3
	3	3	3	3	4	3	3	2	3	1	1	1	2
	1	2	1	2	1	2	5	1	3		3	1	6
		5	5	2		2		1	2	2		2	
	1	2	2		1	2		1					
1	3	29	2	1	8	3	8	1	4	2	2		2
	2	2	2	2	3				2	2	1	1	4
	4	3	4	4	4	4	2	4	3	3	2	2	2

	2	2	3	2	2	2	2	2	3	2	2	2	2
	2	2	2	2	2	2							
				2		2		2	2	2		2	
	2		2		2	2							
2	1	21	2	2	8	3	6	1	2	2	2		4
	2	2	2	2	3				2	2	2		4
	4	4	4	3	4	4	3	3	2	1	1	2	2
	2	2	3	1	3	4	1	1	3	2	2	2	2
				2		2		2	2	2		2	
	2		2		2	2							
1	1	45	2	1	12	2	11	1	1	3	1	2	4
	2	2	2	2	3				2	2	4	3	4
	4	4	4	3	4	4	2	4	3	1	2	2	2
	2	2	2	2	1	2	2	2	3	1	2	2	2
	2	2	2	2	2	2	1		1		1		1
		5		2		2		2	2	2		2	
	2		2		2	2							
2	1	32	2	1	4	4	6	2	2	3	2		2
	2	2	2	2	3				2	2	2	1	3
	4	4	4	3	4	4	4	4	4	1	2	2	2
	2	2	2	2	2	2	2	2	3	2	2	2	2
	2	2	2										
				2		2		2	2	2		2	
	2		2		2	2							
2	1	50	2	1	12	2	11	1	2	3	1	3	3
	2	2	2	2	3				2	4	4	4	3
	5	4	5	4	3	5	3	4	3	3	2	3	3
	2	2	2	2	2	3	2		3	2	2	2	2
				2		2		2	2	2		2	
	2		2		2	2							
2	1	54	1	1	12	2	11	1	2	2	2		2
	2	2	1	2	3				2	2	3	4	4
	4	4	4	4	3	4	3	3	3	1	2	2	2
	2	2	2	2	2	2	2	2	2	1	1	2	2
	2	2	2	2	2	2	1	1	1	1	1	1	1
	1	5	5	2		2		2	2	2		2	
	2		2		2	2							
1	1	28	1	1	12	2	11	1	2	3	1	1	3
	2	1	2	2	2				3	4	3	3	2
	4	3	3	3	3	2	3	4	2	1	5	4	4
	4	4	1	3	3	2	2	3	1	1	2	2	2
	2		2		2		1		1		1		1
		5	5	2		2		2		2			
	2				2								
1	1	38	2	1	12	1	11	1	3	4	2		2
	2	2	2	2	3				3	3	1	3	2
	4	3	3	4	2	4	3	3	3	2	4	3	4
	3	3	3	2	2	2	2	3	2	1	2	2	2
	2	2	2	2	2	2							
				2		2		2	2	2		2	
	2		2		2	2							
1	1	38	2	1	12	2	11	2	1	3	1	1	3
	2	2	2	2	3				2	2	3	2	3
	4	3	4	3	3	3	4	4	3	1	2	3	2

	2	2	2	2	4	2	2	2	3	2	2	2	2
				2		2		2	2	2		2	
	2		2		2	2							
2	1	35	2	1	12	2	11	2	3	3	1	1	3
	2	2	2	2	2				2	2	3	2	4
	4	4	4	4	4	4	3	4	4	2	2	2	2
	2	2	2	2	2	2	2	2	2	2	2	2	2
	2	2	2	2	2	2							
				2		2		2	2	2		2	
	2		2		2	2							
2	1	40	1	1	12	2	11	1	3	3	1	1	3
	2	2	2	2	3				2	2	1	2	3
	4	4	4	4	4	4	3	4	4	1	2	2	2
	2	2	2	2	2	3	2	2	3	2	2	2	2
				2	0	2	0	2	2	2	0	2	0
	2	0	2	0	2	2							
1	1	39	2	1	12	2	11	1	2	3	1	1	3
	2	2	2	2	3				1	2	4	4	4
	5	4	4	4	4	4	4	4	4	1	2	2	2
	2	1	2	1	2	2	1	2	3	2	2	2	2
				2		2		2	2	2		2	
	2		2		2	2							
2	1	39	3	1	6	2	11	1	1	3	2		2
	2	2	2	2	2				2	2	2	2	4
	4	4	4	4	4	4	3	3	4	2	2	2	2
	2	2	2	2	2	2	2	2	4	2	2	2	2
				2		2		2	2	2		2	
	2		2		2	2							
2	1	31	2	3	12	2	11	2	1	3	2		1
	2	2	2	1	2				2	2	2	2	3
	4	3	3		3	4	3	4	4	1	4	3	2
	3	2	2	3	2	2	1	3	2	1	2	1	2
	1	2	1	2	2	2	1		2		1		4
	6	4	6	2		2		2	2	2		2	
	2		2		2	2							
2	2	23	2	2	9	3	2	1	1	2	2		6
	2	1	1	1	3	1	4	1	1	1	4	2	5
	4	4	4	4	4	4	3	4	3	1	2	2	2
	1	2	2	1	1	2	2	1	3	1	1	1	1
	1	2	2	2	2	2	1						
				2		2		2	2	2		2	
	2		2		2	2							
1	1	19	2	2	9	3	2	1	3	2	2		5
	2	2	2	2	3				2	2	2	1	2
	3	3	2	3	3	2	3	3	2	3	2	2	2
	3	2	3	2	2	3	3	2	3	2	2	2	2
				2		2		2	2	2		2	
	2		2		2	2							
1	1	40	2	2	1	1	11	1	2	4	1	2	5
	2	1	2	2	3				2	2	2	3	3
	4	3	3	3	3	5	4	4	3	2	3	2	3

	2	2	3	2	2	4	2	2	3	2	2	2	2
				2		2		2	2	2		2	
	2		2		2	2							
2	1	48	3	1	3	1	11	1	5	4	1	3	4
	2	2	2	2	3				2	2	1	2	5
	5	4	4	3	4	5	5	4	4	2	2	2	2
	2	1	2	2	2	3	2	2	2	2	2	2	2
	2	2											
2	1	48	3	1	3	1	11	1	5	4	1	3	4
	2	2	2										
										2	2	2	2
	2	2	2	2	2	2							
				2		2		2	2	2		2	
	2		2		2	2							
1	1	49	2	1	7	1	11	1	2	2	1	4	3
	2	2	2	2	3	6	5		2	2	1	1	4
	5	4	4	3	3	4	3	3	4	1	1	2	2
	2	2	2	2	1	3	2	2	3	1	2	2	2
				2		2		2	2	2		2	
	2		2		2	2							
1	1	28	5	1	11	3	8			1	1	3	5
	2	2	2	2	3				2	1	2	2	3
	4	4	4	4	5	5	3	2	3	2	2	3	3
	1	2	1	2	1	2	2	3	3	2	2	2	2
				2		2		2	2	2		2	
	2		2		2	2							
2		22	2	2	6	3	4	1	2	2	2		4
	2	2		2	3				3	4	1	1	3
	3	3	3	2	3	2	2	4	4	3	2	2	3
	3	3	5	1	3	2	2	3	3	2	2	2	2
				2		2		2	2	2		2	
	2		2		2	2							
2	1	48	2	1	12	2	11		1	3	1	2	4
	2	2	2	2	3				2	2	2	2	4
	4	4	4	3	4	5	3	3	3	1	2	2	2
	2	2	2	2	2	3	2	2	3	2	2	2	2
				2		2		2	2	2		2	
	2		2		2	2							
2	1	54	2	1	12	1	11	1	2	4	1	4	3
	2	2	2	2	3				2	2	2	2	4
	5	4	4	4	4	4	4	4	3	2	2	2	2
	2	2	2	2	2	3	5	2	3	2	2	2	2
				2		2		2	2	2		2	
	2		2		2	2							
2	1	32	2	1	12	2	11	1	2	3	1	1	3
	2	1	2	1	2				2	3	3	2	2
	4	2	3	4	3	4	3	4	3	1	4	3	2

	3	3	2	4	1	3	3	3	2	1	2	1	2
	1	2	1	2	2	2	4		1		2		4
		5		2		2		2	2	2		2	
	2		2		1	2							
1	1	33	1	2	12	2	11	2	1	2	1	1	2
	2	2	2	2	3				2	4	2	2	2
	4	3	4	4	3	1	2	3	4	1	2	4	3
	4	4	3	3	2	3	3	2	1	1	2	2	2
				2		2		2	2	2		2	
	2		2		2	2							
1	1	40	2	2	12	2	11	1	2	3	1	1	4
	2	2	2	2	3				2	2	2	1	4
	5	4	4	4	4	4	4	4	4	1	2	1	2
	2	2	2	2	2	3	2	2	3	1	2	2	2
				2		2		2	2	2		2	
	2		2		2	2							
2	1	48	2	3	12	2	11		2	3	1	2	2
	2	2	2	2	3				1	2	1	2	5
	5	5	5	4	5	5	5	5	4		1	1	1
	1	1	1	1	3	1	1	1	4	1	2	2	2
				2		2		2	2	2		2	
	2		2		2	2							
2	1	40	2	1	12	2	11	2	1	3	1	1	3
	2	2	2	2	3				2	2	1	2	4
	4	3	4	3	3	4	3	3	3	1	2	2	2
	2	2	2	3	3	4	2	2	3	2	2	2	2
				2		2		2	2	2		2	
	2		2		2	2							
2	1	39	2	1	12	2	11	1	2	4	1	2	4
	2	2	2	2	3				2	2	1	1	4
	5	4	4	4	4	5	4	4	3	2	2	2	2
	2	2	2	2	2	2	2	2	3	2	2	2	2
				2		2		2	2	2		2	
	2		2		2	2							
2	1	40	2	1	12	2	11	1	1	3	2		2
	1	2	2	2	3				2	2	3	4	4
	5	4	5	2	4	5	5	4	4	2	3	2	1
	1	1	1	2	1	3	1	2	3	2	2	2	2
				2		2		2	2	2		2	
	2		2		2	2							
1	1	43	2	1	12	2	11	1	4	3	1	3	8
	2	2	2	2	3				2	2	1	3	4
	5	4	5	5	3	5	4	4	4	2	2	3	1
	1	2	2	4	1	4	3	2	3	2	2	2	2
				2		2		2	2	2		2	
	2		2		2	2							
1	1	43	2	1	12	2	11		1	4	1	2	4
	1	2	2	2	3				2	2	3	1	3
	5	3	4	4	3	5	3	4	3	2	3	2	2

	2	2	2	2	2	3	1	2	4	2	2	2	2
				2		2		2	2	2		2	
	2		2		2	2							
2	1		2	1	12	2	11	2	2	2	1	4	3
	2	2	2	2	3				2	2	2	1	4
	4	3	4	4	3	5	3	4	3	1	4	2	2
	2	2	2	2	1	2	1	2	4	1	2	2	2
	1	2	2	2	2	2							
				2		2		2	2	2		2	
	1		2		2	2							
1	1	34	2	4	12	2	11		2	2	1	1	
	2	2	2	2	3				2	2	3	2	4
	4	3	4	4	4	4	3	4	4	1	3	2	2
	2	2	2	3	2	2	3	2	2	2	2	2	2
				2		2		2	2	2		2	
	2		2		2	2							
1	1	41	2	1	12	2	11	1	1	4	1	2	4
	2	2	2	2	3				1	1	1	1	4
	5	5	5	4	4	5	4	4	4	2	1	1	1
	1	1	1	1	1	2	1	2	4	2	2	2	2
				2		2		2	2	2		2	
	2		2		2	2							
2	1	32	1	1	12	2	11	3		2	1	3	5
	1	2	2	2	3				3	3	1	2	3
	3	4	4	3	5	2	1	3	2	2	3	2	1
	3	4	3	4	2	5	2	2	3	2	2	2	2
				2		2		2	2	2		2	
	2		2		2	2							
1	1	31	2	1	12	2	11	1	2	2	2		2
	2	2	2	2	3				1	1	1	1	4
	5	4	4	4	5	5	3	4	3		2	1	1
	1	2	2	3	2	4	1	2	4	1	2	2	2
				2		2		2	2	2		2	
	2		2		2	2							
2	1	48	3	1	12	2	11	1	1	3	1	3	5
	2	2	2	2	3				2	3	3	2	4
	5		4	3	4	3	3	4	3	1	3	2	2
	2	2	3	3	4	3	3	2	3	2	2	2	2
				2		2		2	2	2		2	
	2		2		2	2							
1	1	47	1	1	12	2	11		4	2	1	2	8
	2	2	2	2	3				2	4	2	3	3
	4	3	3	3	3	3	2	3	4	2	4	2	2
	3	3	2	2	2	3	2	2	3	1	2	2	2
				2		2		2	2	2		2	
	2		2		2	2							
1	1	38	2	1	12	2	11		2	3	1	1	3
	2	2	2	2	3				2	2	4	2	4
	4	4	4	3	4	4	4	4	4	2	2	2	1

	1	1	1	1	1	3	2		3	2	2	2	2
				2		2		2	2	2		2	
	2		2		2	2							
2	1	37	2	1	12	1	11	3		3	1	2	4
	2	2	2	2	3				2	2	2	2	4
	4	4	4	3	4	4	3	4	3	1	2	3	2
	2	3	2	2	2	3	2	2	4	2	2	2	2
				2		2		2	2	2		2	
	2		2		2	2							
2	1	43	1	1	12	1	11	3		3	1	2	4
	2	1	2	2	3				2	2	1	3	4
	5	4	4	5	4	4	3	5	3	2	3	3	3
	1	2	1	3	2	4	1	3	3	2	2	2	2
				2		2		2	2	2		2	
	2		2		2	2							
1	1	45	2	2	10	1	11	1	1	3	1	1	5
	2	2	2	2	3				2	3	2	2	4
	5	3	4	4	4	3	3	4	3	2	3	2	2
	3	2	3	2	1	3	3	2	3	2	2	2	2
				2		2		2	2	2		2	
	2		2		2	2							
1		39	1	1	7	1	11	1	4	4	1	3	5
	2	2	2	2	3				2	3	3	2	4
	4	4	5	4	4	5	3	4	3	2	2	2	1
	2	2	1	2	1	2	1	1	3	2	2	2	2
				2		2		2	2	2		2	
	2		2		2	2							
2	3	57	2	6	12	1	11		1	3	1	3	1
	2	1	2	2	3				2	3	2	3	3
	5	4	3	3	3	4	3	4	2	1	2	2	2
	3	3	3		4	3	2	3	3	2	2	2	2
				2		2		2	2	2		2	
	2		2		2	2							
1	1	25	2	2	9	1	11	1	3	3	2		1
	2	2	2	1	1	3	2	1	3	4	4	2	2
	4	3	3	4	3	2	3	3	2		3	3	2
	3	2	2	2	2	3	4	3	1	2	2	1	2
	1	2	2	2	2	2	2		1		2		1
		3		1	3	2		1	2	1	1	2	
	1	2	2		1	2	2018	1	1	2011	3	1	
	2005	2	1										
2	1	51	3	1	12	1	11		1	3	1	2	4
	2	2	2	2	3				2	2	2	2	4
	4	4	4	4	4	4	3	4	3	2	1	2	2
	2	2	2	3	3	2	2	2	3	2	2	2	2
				2		2		2	2	2		2	
	2		2		2	2							
1	1	54	2	2	7	1	11		1	3	2		5
	2	2	2	2	3				4	2	1	2	3
	5	3	2	4	4	4	2	3	3	2	4	2	2

	2	3	2	2	4	4	2	3	3	2	2	2	2
				2		2		2	2	2		2	
	2		2		2	2							
2	1	42	1	3	12	1	11	1	1	4	2		1
	2	2	2	2	3				5	5	1	1	5
	5	5	5	5	5	5	5	5	5		1	1	1
	1	1	1	1	1	3	2	2	5	2	2	2	2
				2		2		2	2	2		2	
	2		2		2	2							
2	1	55	2	1	12	1	11	1	2	3	1	3	1
	2	2	2	1	2				2	2	2	1	3
	3	2	4	4	3	4	2	2	3	1	3	2	2
	4	3	5	4	2	5	2	4	2	1	2	1	2
	1	2	2	2	2	2	3		3		3		1
		5		2		2		2	2	2		2	
	1		2		2	2							
1	1	52	2	1	9	1	11	1	1	3	1	2	6
	2	2	2	2	3				1	2	2	3	3
	5	4	4		4	4	3	4	3	1	3	2	2
	2	2	2	2	1	2	2	1	3	2	2	2	2
				2		2		2	2	2		2	
	2		2		2	2							
2	1	46	2	6	12	1	11	3		4	1	2	3
	2	1	2	2	3				2	2	3	2	4
	5	4	5	2	5	1	3	4	5	1	2	2	3
	3	3	1	2	4	4	2	2	5	2	2	2	2
				2		2		2	2	2		2	
	2		2		2	2							
2	1	38	3	1	8	1	11	1	2	3	1	1	3
	2	2	2	2	3				2	2	1	1	4
	5	4	5	4	5	5	3	4	4	2	2	2	1
	2	1	2	2	2	3	3	2	3	2	2	2	2
				2		2		2	2	2		2	
	2		2		2	2							
2	1	57	1	1	12	1	11	2	1	4	2		2
	2	2	2	2	3				1	1	1	1	4
	5	4	5	5	5	5	5	4	5	2	2	1	1
	1	1	3	2	1	1	1	1	4	2	2	2	2
				2		2		2	2	2		2	
	2		2		2	2							
1	1	34	1	1	12	1	11	1	1	3	1	2	4
	2	2	2	2	3				3	3	2	1	3
	5	3	4	4	3	4	4	5	2	2	4	3	3
	2	2	1	2	2	3	2	2	2	2	2	2	2
				2		2		2	2	2		2	
	2		2		2	2							
1	1	33	1	1	8	1	11	1	3	3	1	1	3
	2	2	2	2	3				2	2	2	2	2
	5	5	3	3	3	3	3	5	2	1	5	5	2

	4	4	3	5	5	4	1	2	5	1	1	2	2
				2		2		2	2	2		2	
	2		2		2	2							
1	1	53	2	2	7	1	11		1	3	1	3	3
	2	2	2	2	3				2	4	4	3	4
	5	4	4	4	3	3	2	3	3	1	4	3	3
	3	2	4	4	3	3	3	3	2	1	2	2	2
				2		2		2	2	2		2	
	2		2		2	2							
2	1	38	2	1	12	1	11		2	3	1	3	5
	2	2	1	2	3				1	2	1	1	4
	4	3	4	4	4	4	3	3	3	1	3	2	2
	2	3	3	4	2	2	2	2	2	1	2	1	2
	1	2	2	2	2	2	5		2		4		1
		5		2		2		2	2	2		2	
	2		2		2	2							
2	1	41	3	1	1	1	11	1	1	4	1	1	3
	2	2	2	2	3				2	2	1	2	4
	5	5	4	3	4	4	3	4	4	2	3	2	2
	1	3	2	3	2	3	3	2	2	2	2	2	2
				2		2		2	2	2		2	
	2		2		2	2							
1	1	45	2		12	1	11	1	2	3	2		1
	2	2	2	2	3				2	3	3	3	3
	4	3	4	5	3	3	3	4	3	3	3	3	3
	3	3	3	3	3	3	3	3	4	2	2	2	2
				2		2		2	2	2		2	
	2		2		2	2							
1	1	56	1	1	12	1	11	3		3	2		3
	2	2	2	2	3				2	3	3	3	4
	4	4	3	3	3	4	4	3	3	1	3	3	3
	2	2	2	2	2	2	2	3	3	2	2	2	2
				2		2		2	2	2		2	
	2		2		2	2							
2	1	53	1	1	12	1	11		2	3	2		2
	2	2	2	2	3				2	3	1	3	5
	5	4	4	4	5	4	4	3	5	1	2	2	2
	2	2	2	2	2	4	4	2	3	2	2	2	2
				2		2		2	2	2		2	
	2		2		2	2							
1	1	34	1	1	12	1	11		4	3	1	2	4
	2	1	2	1	1	3	2	1	2	4	2	3	3
	4	3	2	4	3	2	3	3	2	1	4	4	2
	4	4	4	4	2	2	2	2	1	1	1	1	2
	2	2	1	2	2	2	4		5		3		1
		5		2		2		1	2	2		2	
	1		2		2	2							
2	1	58	2	1	7	1	11	2	1	3	1	3	2
	2	2	2	2	3				2	1	2	1	3
	5	4	5	5	5	5	2	4	3	2	2	2	2

	1	2	1	2	2	3	2	3	2	2	2	2	2
				2		2		2	2	2		2	
	2		2		2	2							
1	1	44	1	1	9	1	11	1	4	3	1	2	4
	2	2	1	2	3				1	1	1	1	4
	5	4	4	4	4	4	3	5	5	2	2	1	1
	1	3	2	2	1	2	1	1	3	1	2	2	2
				2		2		2	2	2		2	
	2		2		2	2							
1	1	52	1	4	1	1	11		2	4	1	2	2
	2	2	2	2	3				2	2	4	3	3
	5	4	4	3	4	4	4	4	3	3	1	2	2
	2	2	3	3	1	2	2	2	3	2	2	2	2
				2		2		2	2	2		2	
	2		2		2	2							
1	1	49	1	2	12	1	11		2	4	2		1
	2	2	2	2	3				2	2	2	3	3
	4	3	4	4	4	3	4	4	3	3	3	2	2
	2	3	5	3	1	2	2	4	3	2	2	2	2
				2		2		2	2	2		2	
	2		2		2	2							
1	1	51	3	5	12	1	11		2	3	1	2	4
	2	2	2	2	3				1	2	2	1	5
	5	4	4	4	4	4	3	4	4	2	1	2	2
	1	1	1	1	1	3	2	2	4	2	2	2	2
				2		2		2	2	2		2	
	2		2		2	2							
2	1	57	2	4	12	1	11	2	1	3	1	2	3
	2	2	2	2	3				2	3	3	4	3
	4	3	3	4	3	3	3	4	3	1	3	4	3
	2	3	3	3	2	3	2	2	3	2	2	2	2
				2		2		2	2	2		2	
	2		2		2	2							
1	1	44	2	1	7	1	11		1	3	1	2	4
	2	2	2	2	3				2	3	4	4	3
	4	3	5	4	3	4	4	3	2	1	3	2	2
	2	3	3	3	2	4	3	2	3	1	2	2	2
				2				2	2	2		2	
	2		2		1	2							
2	1	60	1	1	12	1	11	3		4	2		3
	2	2	2	1	2				1	1	1	1	4
	5	4	4	4	3	5	4	5	3	2	4		3
	2	2	2	3	1	3		1	3	1	2	2	2
				2		2		2	2	2		2	
	2		2		2	2							
2	2	40	2	2	12	1	11	3		3	2		1
	2	1	2	1	1	3	1	1	3	2	2	2	3
	2	5	3	4	2	3	3	4	3	2	4	4	4

	5	3	2	2	2	2	2	4	1	1	1	1	1
	1	2	1	2	1	2	3	1	5	3	6	6	
	4	5	5	1	3	2		1	1	1	1	2	
	1	1	2		1	2	2020	1		2003	4	3	
	2003	4	3										
1	1	42	2	1	12	1	11		1	3	2		2
	2	2	2	2	3				2	2	2	1	3
	4	4	4	4	4	4	3	4	3	2	3	2	2
	2	3	3	3	2	4	2	2	3	2	2	2	2
				2		2		2	2	2		2	
	2		2		2	2							
1	1	35	2	1	12	2	11	1	1	3	2		2
	1	1	2	2	3				4	4	2	3	4
	4	2	5	5	2	5	3	5	5	1	2	3	2
	3	1	2	3	1	2	1	3	2	1	2	1	2
	2	2	2	2	2	2	1		1		1		1
		5		2		2		2	2	2		2	
	2		2		2	2							
2	1	45	2	4	12	2	11		3	3	1	1	2
	1	2	2	1	2				3	4	4	4	3
	4	2	3	4	2	5	2	5	5	1	4	3	2
	2	2	4	4	1	2	2	2	3	1	2	1	2
	2	2	2	2	2	2	2		1		2		5
		5		2		2		2	2	2		2	
	2		2		2	2							
2	1	32	1	1	12	1	11	3		3	2		1
	2	2	2	1	2				2	2	1	1	4
	5	4	4	5	3	4	5	5	4	2	4	2	3
	2	2	1	1	2	2	4	1	3	1	2	1	2
	1	2	1	2	2	2	2		1		6		5
		5		1	1	2		2	2	2		2	
	2		2		2	2	2010	5	3				
2	1	42	1	1	12	1	11	3		3	2		2
	2	1	2	2	3				3	3	3	2	2
	4	4	3	3	3	4	3	4	3	1	4	3	3
	3	2	3	3	4	4	2	3	1	2	2	2	2
				2		2		2	2	2		2	
	2		2		2	2							
1	1	30	2	1	1	5	11	2	1	3	2		2
	2	2	2	1	2				3	3	1	1	2
	3	3	2	3	2	3	3	3	2	2	5	4	4
	4	3	4	2	3	3	3	1	1	1	1	1	1
	2		2	2	2		1		1		2		1
		5		2	0	2	0	2	2	2	0	2	0
	2	0	2	0	2	2							
2	1	46	2	1	12	1	11	1	1	3	1	2	4
	2	2	2	2	3				2	2	1	2	4
	5	5	4	5	5	5	5	4	4	1	2	2	2
	2	2	2	2	1	2	1	2	3	2	2	2	2
				2		2		2	2	2		2	
	2		2		2	2							
2	1	39	1	2	12	1	11	3		3	2		1
	2	2	2	1	2				2	2	1	1	3
	4	3	4	4	3	5	5	5	3	2	2	2	2

	2	3	2	3	2	2	1	2	5	1	1	1	1
	1	1	1	2	1	2	1	1	1	1	1	1	1
	1	5	5	1	1	2		2	2	2		2	
	2		2		2	2							
1	1	59	1	4	7	1	11	1	2	3	1	3	1
	2	2	2	2	3				3	3	4	3	2
	5	4	5	3	3	4	4	5	3	3	1	2	2
	1	1	2	1	3	2	1	2	4	2	2	2	2
				2		2		2	2	2		2	
	2		2		2	2							
2	1	38	1	1	1	1	11	2	1	4	1	2	4
	2	2	2	1	2	6			2	4	4	2	2
	4	2	3	3	3	2	3	3	2	3	4	4	4
	4	4	2	4	3	3	4	3	5	1	2	1	2
	2	2	1	2	2	2	1		1		5		4
		5		2				2	2	2		2	
	2		2		2	2							
2	1	45	3	1	1	1	11	2	1	4	1	3	5
	2	2	2	2	3				1	1	1	1	4
	5	5	5	4	5	5	3	4	5	2	1	1	1
	1	1	1	2	1	1	1	3	3	2	2	2	2
	2	2	2	2	2	2	0		0		0		0
		0		2	0	2	0	2	2	2	0	2	0
	2	0	2	0	2	2							
1	1	39	2	1	1	1	11	1	2	3	1	2	4
	2	2	2	2	3				2	3	1	1	3
	4	3	3	3	3	3	3	4	3	2	2	2	2
	3	3	3	3	1	3	2	3	3	1	2	2	2
	2	2	2	2	2	2	0		0		0		0
		0		2		2		2	2	2		2	
	2		2		2	2							
1	2	41	2	1	1	1		3		3	2		2
	2	2	2	2	3				2	2	1	2	4
	5	4	3	3	3	5	4	4	4	1	2	2	2
	1	2	1	1	3	3	2	4	3	2	2	2	2
	2	2	2	2	2	2	0		0		0		0
		0		2		2		2	2	2		2	
	2		2		2	2							
1	1	37	1	1	12	2	11	2	2	3	2	2	5
	2	2	2	2	3				3	4	2	3	3
	4	3	3	3	3	3	3	4	2	2	2	4	2
	3	4	4	2	3	2	4	3	3	1	2	2	2
	2	0	2	2	2	2	0		0		0		0
		0		2		2		2	2	2		2	
	2		2		2	2							
1	1	34	2	1	1	1	11	1	2	4	2	1	3
	2	2	2	1	2				2	3	1	4	3
	4	3	4	4	5	4	5	5	3	2	3	2	3
	2	2	5	2	1	1	1	4	3	1	2	1	2
	2	2	2	2	2	2	1		1		3		1
		4		1	1	2		2	2	2		2	
	1	1	2		1	2	38681	1	3	38681	1	3	
	38681	1	3										
1	1	40	2	4	1	1	11	2	1	4	2	1	5
	2	2	1	1	2				2	3	2	3	3
	3	2	2	4	2	2	3	4	4	2	4	4	4

	4	3	2	3	2	2	1	4	5	1	1	2	2
	2	2	2	2	2	2	0		0		0		0
		0		2		2		2	2	2		2	
	2		2		2	2							
2	1	44	1	1	8	2	11	1	1	3	2	3	5
	2	2	2	1	1	3	2	1	2	2	2	2	3
	4	4	4	3	5	5	3	3	2	1	2	2	2
	1	3	3	1	4	4	3	3	3	1	2	1	2
	1	2	2	2	2	2	1		1		2		1
		4		2		2		1	2	2		2	
	2		2		2	2							
1	1	40	2	1	4	1	11	1	4	4	2	2	3
	2	2	2	2	3				2	1	2	2	2
	4	3	3	2	3	4	4	4	1	2	2	3	4
	2	4	4	4	3	2	2	4	1	1	1	1	2
	1	2	2	2	2	2	3		2		1		4
		3		2		2		2	2	2		2	
	2		2		2	2							
1	1	20	2	2	7	3	5	3		1	1		6
	2	2	2	1	1		10	1	4	5	3	3	1
	2	2	2	3	2	2	1	3	2	1	5	5	4
	5	4	4	4	5	5	3	4	1	1	1	1	1
	1	2	1	2	1	2	5		5		6		4
		5		1	2	2		1	1	1	10	2	
	1	1	2		1	2	2019	2	2	2019	2	2	
	2012	2	1										
2	1	20	2	2	7	3	5	1	1	1	1		5
	2	2	2	1	2				2	3	1	2	3
	2	2	3	3	3	5	3	3	2	3	4	3	3
	3	4	1	5	1	3	3	3	5	1	2	1	2
	1	2	1	2	2	2			5		2		1
		5		2		2		1	2	2		2	
	2		2		2	2							
2	1	43	1	4	8	1	11	1	3	3	1		1
	2	2	2	2	3				2	4	3	3	3
	4	3	4	4	3	4	3	3	3	1	4	3	3
	2	2	2	3	2	3	2	3	3	1	2	2	2
				2		2		2	2	2		2	
	2		2		2	2							
1	3	25	2	2	7	3	5	1	1	1	1		6
	2	2	2	1	2				3	4	2	3	3
	1	1	2	3	2	2	1	2	2	1	4	5	4
	5	4	2	5	3	4	3	4	2	1	1	1	2
	1	2	1	2	1	2	3		4		4		1
		5		1	1	2		2	2	2		2	
	1	3	2		1	2	2021	1	1	2021	1	1	
	2021	1	1										
1	1	23	3	3	7	3	8	3		1	2	2	3
	2	2	2	2	3				2	4	3	4	5
	4	2	3	3	2	4	2	3	3	4	5	4	2
	1	1	2	4	2	4	4	3	3	1	2	1	2
	1	2	1	2	1	2	4		3		6		1
		5		2		2		2	2	1	1	2	
	2		2		1	2							
1	1	26	2	1	7	3	8	1	1	2	2	1	4
	2	2	2	2	3				3	3	4	3	5
		3	4	3	3	4	3	3	4	1	2	2	2

	2	3	2	3	2	2	3	3	3	2	2	2	2
				2		2		2	2	2		2	
	2		2		2	2							
1	1	24	2	2	7	3	7			1	1		6
	2	2	2	2	3				3	2	1	2	4
	4	4	3	3	3	2	3	3	3	2	5	3	2
	3	3	2	3	2	3	3	2	3	2	2	2	2
				2		2		2	2	2		2	
	2		2		2	2							
1	1	24	2	2	7	3	7	3		2	1		2
	2	1	2	1	1	2	2	1	3	5	3	5	2
	3	2	2	1	1	1	1	2	2	3	5	5	5
	5	4	5	5	1	5	3	3	5	1	1	1	2
	1	2	1	2	1	2	3	1	2	1	1	1	1
		5		2		2		1	2	2		2	
	2		2		1	1							
1	1	21	2	2	7	3	3	3		2	1		6
	2	2	2	2	3				3	3	2	1	3
	5	2	3	3	2	3	2	3	3	3	3	4	4
	3	3	2	2	3	4	4	3	1	2	2	2	2
				2		2		1	2	2		2	
	2		2		2	2							
1	3	19	2	2	7	3	3	1	1	1	1		4
	2	2	2	2	3				3	3	3	1	3
	2	2	2	4	2	2	3	3	2	1	5	4	3
	5	4	3	4	3	5	2	2	5	1	2	1	2
	1	2	1	2	1	2	4		5		4		1
		5		2		2		1	2	2		2	
	2		2		1	2							
2	1	21	2	2	7	3	4	3		1	1		8
	2	2	2	2	3				2	2	1	1	4
		3	3	3	5		1	2	2	3	3	2	2
	2	2	3	5	2	2	3	3	3	2	2	2	2
				2		2		2	2	2		2	
	2		2		2	2							
1	2	30	2	1	8	1	5	3		3	1		
	2	1	2	2	1	4	2	1	2	2	1	1	3
	5	4	4	4	3	5	3	4	3	2	2	2	2
	2	2	2	2	2	2	2	2	3	2	2	2	2
				2		2		2	2	2		2	
	2		2		2	2							
1	1	31	2	1	1	1	11	1	1	4	1		2
	2	2	2	2	3				2	2	1	1	4
	5	4	4	4	4	5	5	4	4	2	2	2	1
	1	2	2	2	2	2	1	2	3	2	2	2	2
				2		2		2	2	2		2	
	2		2		2	2							
1	1	38	2	1	2	1	4	3		4	2	2	3
	2	2	2	2	3				2	2	2	1	4
	4	4	4	4	4	3	4	4	3	1	2	2	2

2	2	3	2	3	2	2	2	3	2	2	2	2
2	2	2	2	2	2							
			2				2	2	2		2	
2		2		2	2							
